# Structure-Based Screening of *Plasmodium berghei* Glutathione S-Transferase Identifies CB-27 as a Novel Antiplasmodial Compound

**DOI:** 10.3389/fphar.2020.00246

**Published:** 2020-03-17

**Authors:** Emilee E. Colón-Lorenzo, Daisy D. Colón-López, Joel Vega-Rodríguez, Alice Dupin, David A. Fidock, Abel Baerga-Ortiz, José G. Ortiz, Jürgen Bosch, Adelfa E. Serrano

**Affiliations:** ^1^Department of Microbiology and Medical Zoology, University of Puerto Rico School of Medicine, San Juan, PR, United States; ^2^Department of Biochemistry and Molecular Biology, Johns Hopkins Bloomberg School of Public Health, Baltimore, MD, United States; ^3^Department of Microbiology and Immunology, Columbia University Medical Center, New York, NY, United States; ^4^Division of Infectious Diseases, Department of Medicine, Columbia University Medical Center, New York, NY, United States; ^5^Department of Biochemistry, University of Puerto Rico School of Medicine, San Juan, PR, United States; ^6^Department of Pharmacology and Toxicology, University of Puerto Rico School of Medicine, San Juan, PR, United States; ^7^Division of Pediatric Pulmonology and Allergy/Immunology, Case Western Reserve University, Cleveland, OH, United States; ^8^InterRayBio, LLC, Baltimore, MD, United States

**Keywords:** malaria, *Plasmodium berghei*, glutathione S-transferase, drug target, structure-based screening, shape similarity screening, antimalarial drug discovery, predicted pharmacokinetic and toxicity parameters

## Abstract

*Plasmodium falciparum* parasites are increasingly drug-resistant, requiring the search for novel antimalarials with distinct modes of action. Enzymes in the glutathione pathway, including glutathione S-transferase (GST), show promise as novel antimalarial targets. This study aims to better understand the biological function of *Plasmodium* GST, assess its potential as a drug target, and identify novel antiplasmodial compounds using the rodent model *P. berghei*. By using reverse genetics, we provided evidence that GST is essential for survival of *P. berghei* intra-erythrocytic stages and is a valid target for drug development. A structural model of the *P. berghei* glutathione S-transferase (PbGST) protein was generated and used in a structure-based screening of 900,000 compounds from the ChemBridge Hit2Lead library. Forty compounds were identified as potential inhibitors and analyzed in parasite *in vitro* drug susceptibility assays. One compound, CB-27, exhibited antiplasmodial activity with an EC_50_ of 0.5 μM toward *P. berghei* and 0.9 μM toward *P. falciparum* multidrug-resistant Dd2 clone B2 parasites. Moreover, CB-27 showed a concentration-dependent inhibition of the PbGST enzyme without inhibiting the human ortholog. A shape similarity screening using CB-27 as query resulted in the identification of 24 novel chemical scaffolds, with six of them showing antiplasmodial activity ranging from EC_50_ of 0.6–4.9 μM. Pharmacokinetic and toxicity predictions suggest that the lead compounds have drug-likeness properties. The antiplasmodial potency, the absence of hemolytic activity, and the predicted drug-likeness properties position these compounds for lead optimization and further development as antimalarials.

## Introduction

Malaria is currently considered the world’s most devastating parasitic disease. In 2017, the WHO estimated 219 million cases of malaria, representing an increase of 2 million cases from the previous year (World Health Organization [WHO], 2018). Despite significant advances in reducing the incidence and deaths due to malaria worldwide, this progress has plateaued, and in some regions regressed mostly due to the emergence and spread of the *Plasmodium* parasite’s multidrug resistance to front-line drugs, including artemisinin-based combination therapies (Ashley et al., 2014; Fairhurst and Dondorp, 2016). The parasite’s multidrug resistance highlights the urgent need for novel drugs with different and unique mechanism(s) of action and emphasizes the prioritization of targeted antimalarial drug development. A better understanding of the biology of *Plasmodium* parasites should be exploited to identify new drug targets.

Glutathione S-transferase (GST) has been proposed as a target for the development of novel antimalarials (Harwaldt et al., 2002; Fritz-Wolf et al., 2003). The major role of GST is cellular detoxification via conjugation of glutathione (GSH) to endobiotic and xenobiotic compounds, increasing their solubility and facilitating their excretion from the cell (Mannervik et al., 1988; Armstrong, 1997; Salinas and Wong, 1999; Strange et al., 2000). Additional functions of GST include nucleophilic addition of GSH to toxic compounds, reduction of hydroperoxides, and as a carrier protein (ligandins) of specific organic molecules that result in the inactivation and immobilization of these molecules. *Plasmodium* GST can also bind ferriprotoporphyrin IX (FPIX) produced during hemoglobin digestion to mediate its detoxification (Hiller et al., 2006). Eukaryotic organisms usually have multiple GSTs while the human malaria parasite *P. falciparum* and the rodent malaria *P. berghei* have only one cytosolic GST (Harwaldt et al., 2002; Liebau, 2002; Fritz-Wolf et al., 2003), and membrane-bound GST, known as *P. falciparum* exported protein 1 (PF3D7_1121600) (Lisewski et al., 2014, 2018).

The three-dimensional (3D) structure of *P. falciparum* GST (PfGST) (Burmeister et al., 2003; Fritz-Wolf et al., 2003; Perbandt et al., 2004) has been classified as a sigma class GST based on phylogenetic and structural analyses (Colón-Lorenzo et al., 2010). PfGST exists in a dimer-tetramer equilibrium regulated by GSH binding, and similar to other GSTs, only the dimeric form of PfGST is active (Hiller et al., 2006; Tripathi et al., 2007). The dimer-to-tetramer transition (Fritz-Wolf et al., 2003; Hiller et al., 2006; Tripathi et al., 2007; Liebau et al., 2009; Perbandt et al., 2015) is a phenomenon exclusive to *Plasmodium* GSTs that causes enzyme inactivation (Liebau et al., 2009; Tripathi et al., 2009). The primary structural difference between PfGST and other GST structures is the presence of an additional loop in the *Plasmodium* enzyme, connecting the alpha helix 4 to alpha helix 5 (residues 113-120) which are known to be involved in dimer formation and consequently in enzyme activity (Hiller et al., 2006; Liebau et al., 2009). The active site of the GST dimeric form is composed of the G-site that binds GSH and the H-site that binds a variety of substrates. This additional loop, essential for inter-subunit communication, is located at the H-site and is involved in enzyme tetramerization and cooperativity (Liebau et al., 2009). These features led us to predict that small molecules that either target this loop or affect the dimer-tetramer equilibrium could be effective inhibitors of the *Plasmodium* GST and therefore, potential novel antimalarial drugs.

In this report, we used reverse genetics to show the essential role of GST in *P. berghei* blood stages and explore its potential as a drug target. A structure-based screening against *P. berghei* GST (PbGST) using the ChemBridge Hit2Lead library revealed one lead compound, CB-27, exhibiting antiplasmodial activity at the nanomolar range and inhibiting PbGST in a dose-dependent manner. Six additional chemical scaffolds with antiplasmodial activity were also identified in a shape similarity screening using CB-27 as query. Our results showed that these lead compounds do not have toxicity against erythrocytes and display drug-like properties, including intestinal absorption, metabolism in the liver, drug distribution into the brain, and low excretion; and then, have the potential to become drug candidates. These scaffolds represent novel leads for further development as antimalarials targeting the *Plasmodium* GST.

## Materials and Methods

### *P. berghei* Lines and Mice Infection

The *P. berghei* ANKA WT strain reference line 507cl1 (ANKA 507cl1) expressing green fluorescent protein (Janse et al., 2006a, b) and *P. berghei* GFP-Luc_ama__1_ (1037cl1) expressing green fluorescent protein and the firefly luciferase gene (Spaccapelo et al., 2010) were used in this study. *P. berghei* infections were maintained in 6–8 weeks old random-bred Swiss albino CD-1 female mice (Charles River Laboratories, Wilmington, MA, United States) (Peters, 1975; Peters et al., 1977; Peters and Robinson, 1992). All animal procedures were done at the AAALAC accredited University of Puerto Rico-Medical Sciences Campus Animal Resources Center and approved by the Institutional Animal Care and Use Committee (Protocol number 2480108). All mouse work was done in strict accordance with the “Guide for the Care and Use of Laboratory Animals” (National-Research-Council, Current Edition) and regulations of the PHS Policy on Humane Care and Use of Laboratory Animals. Mice were maintained and housed according to NIH and AAALAC regulations and guidelines. Mice were allowed to acclimate for 1 week before the beginning of the studies. The Animal Experiments Committee approved all animal experiments performed at the Leiden University Medical Center of the Leiden University Medical Center (DEC 07171; DEC 10099). The Dutch Experiments on Animal Act is established under European guidelines (EU directive no. 86/609/EEC regarding the Protection of Animals used for Experimental and Other Scientific Purposes).

### DNA Sequencing of the *P. berghei gst* Gene

Genomic DNA (gDNA) and complementary DNA (cDNA) from *P. berghei* ANKA 507cl1 were PCR-amplified using primer design based on the *P. falciparum* and *P. yoelii yoelii gst* genes (GenBank accession numbers: AY014840 and XM_720396). Amplification of the *P. berghei gst* (*pbgst*) gene fragments was carried out under standard conditions using primers described in Supplementary Table S1. The following primer pairs were used: 211/212, 213/214, 213/215, and 211/214. The PCR-amplified products were gel-purified, cloned into TOPO TA vector (Invitrogen^TM^), and transformed into *Escherichia coli* PMC103 competent cells. Purified clones were sequenced using the Applied Biosystems Big Dye Terminator V3.0 sequencing chemistry (Davis Sequencing Inc., CA, United States). Open reading frames for gDNA and cDNA were assembled using Clone Manager Professional (Version 9.1 for Windows, Scientific and Educational Software). The ExPASy Translate tool and ExPASy Compute pI/Mw tool were used to predict the amino acid sequence and molecular weight of *P. berghei* GST, respectively.

### Generation of Knockout Plasmids and Transfections

**FIGURE 1:**
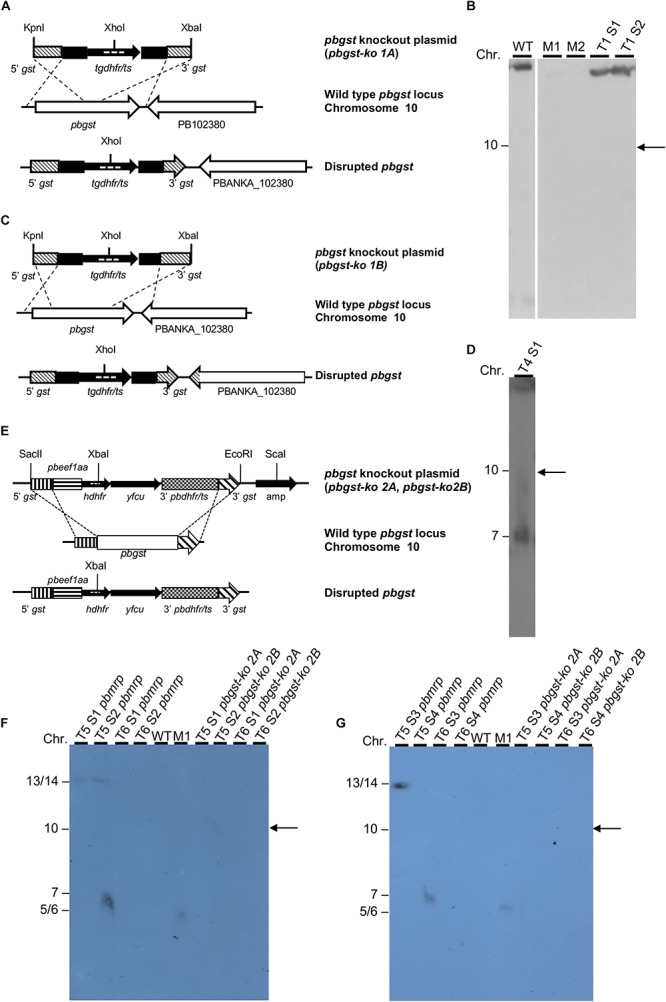
Diagrammatic representation of the *pbgst-ko* plasmids and analysis of potential integration. **(A,C)** Schematic diagram of the *pbgst-ko* plasmids (top), the endogenous *pbgst* locus (center), and the predicted integration (bottom). The plasmids, *pbgst-ko 1A* **(A)** and *pbgst-ko 1B* **(B)**, contained the *tgdhfr*/*ts* selectable marker flanked by the 5′ and 3′ fragments of the *pbgst* gene. The probe used in Southern blot analysis is shown as dashed lines inside the coding region of the *tgdhfr/ts*. **(B,D)** Southern analyses using a *tgdhfr/ts* specific probe. Parasite chromosomes from two independent transfections were separated by CHEF **(B)** or FIGE **(D)**. Hybridization with a *tgdhfr/ts* specific probe shows no integration of the *pbgst-ko 1A* plasmid into the *pbgst* locus **(B)**. Hybridization with a *tgdhfr/ts* specific probe shows integration into chromosome 7 and not into the endogenous *pbgst* locus (chromosome 10, right panel) **(D)**. **(E)** Schematic diagram of the plasmids *pbgst-ko 2A* and *pbgst-ko 2B* (top), the endogenous *pbgst* locus (center), and the predicted integration (bottom). The *pbgst-ko 2* plasmids contained the *hdhfr/yfcu*, positive-negative selectable marker under the control of the *eef1a* promoter. The probe used in Southern blot analysis is shown as dashed lines inside the coding region of *hdhfr*. **(F,G)** Southern analyses using an *hdhfr* specific probe. Hybridization with the *hdhfr* specific probe shows no integration of the corresponding plasmid (lanes 7–10 in **F,G**), *pbgst-ko 2A* or *pbgst-ko 2B*, into the *pbgst* locus (chromosome 10, right arrows) in two independent transfections. As a control, transfection targeting the *pbmrp* (a dispensable gene) shows a successful integration of the *pbmrp-ko* plasmid into chromosome 14 (**F**, lanes 1 and 2 labeled as T5 S1 *pbmrp* and T5 S2 *pbmrp*; and **G**, lane 1 labeled as T5 S3 *pbmrp*). Diagrams are not drawn to scale. M1, *H. wingei* chromosome marker; M2, *S. cerevisiae* chromosome marker; T#S#, transfection number, and sample number.

Two different plasmids (pL0001 and pL0034) were used to create a total of four knockout plasmids (Figure 1) to attempt disruption of the *pbgst* gene. All plasmids were designed to integrate by double crossover recombination guided by 5′ and 3′ targeting regions that were amplified from *P. berghei* ANKA 507cl1 *gst* locus, using primers detailed in Supplementary Table S2.

The *pbgst* knockout plasmids (*pbgst-ko 1A* and *pbgst-ko 1B*) have the pL0001 plasmid backbone (MRA-770, BEI Resources), which contains the *Toxoplasma gondii* dihydrofolate reductase/thymidylate synthase (*tgdhfr/ts*) selection marker cassette. For plasmid design, the *pbgst* DNA sequence (gene identifier in PlasmoDB as PB301263.00.0) was retrieved from PlasmoDB as an incomplete sequence. The 5′ and 3′ targeting regions of the *pbgst* gene were PCR-amplified using the corresponding primer pairs that produced DNA fragments for *pbgst-ko 1A* and *1B* plasmids as detailed in Supplementary Table S2. The 5′ and 3′ targeting regions were independently cloned into the TOPO TA vector (Invitrogen^TM^), digested and then cloned into the pL0001-digested plasmid. The 5′ targeting region was digested with *Kpn*I/*Hin*dIII and cloned into *Kpn*I/*Hin*dIII pL0001-digested plasmid. The 3′ targeting region was digested with *Bam*HI/*Xba*I and cloned into *Bam*HI/*Xba*I pL0001-5′ *gst*-digested plasmid to create the final *pbgst-ko* plasmid. For transfection, the resulting *pbgst-ko* plasmids (*pbgst-ko 1A* and *1B*) were linearized using *Kpn*I/*Xba*I and transfected independently into *P. berghei* ANKA 507cl1 purified schizonts.

The *pbgst-ko 2A* and *2B* plasmids were generated using the pL0034 plasmid backbone (MRA-849, BEI Resources), which contains the positive-negative selectable marker cassette *human dihydrofolate reductase/yeast cytosine deaminase* and *uridyl phosphoribosyl transferase* (*hdhfr/yfcu*) under the control of the constitutive *eukaryotic elongation factor 1A* promoter. For plasmid design, the complete DNA sequence of the *pbgst* gene was obtained by sequencing the gene. For *pbgst-ko 2A* and *2B* plasmids, the 5′ and 3′ targeting regions of the *pbgst* gene were PCR-amplified using the corresponding primer pairs that produced DNA fragments as detailed in Supplementary Table S2. The 5′ and 3′ targeting regions were independently cloned into the TOPO TA vector (Invitrogen^TM^), digested and cloned into the pL0034-digested plasmid. The 5′ targeting region was digested with *Sac*II/*Pst*I and cloned into *Sac*II/*Pst*I pL0034-digested plasmid. The 3′ targeting region was digested with *Eco*RV/*Eco*RI and cloned into *Eco*RV/*Eco*RI pL0034-5′*gst*-digested plasmid to create the final *pbgst-ko* plasmid. For transfection, the resulting *pbgst-ko 2A* and *2B* plasmids were linearized using *Sac*II/*Eco*RI/*Sca*I and transfected independently into *P. berghei* ANKA 507cl1 purified schizonts.

*P. berghei* transfections were accomplished by electroporation of purified schizonts using the Amaxa^®^ Nucleofector^®^ Technology (Lonza) as described previously (Janse et al., 2006a, b). Parasites transfected with *pbgst*-*ko 1A* and *1B*, containing the *tgdhfr/ts* selectable marker, were selected with pyrimethamine as previously described (Janse et al., 2006a, b). Transfection with *pbgst*-*ko 2A* and *2B*, which contains the *hdhfr* selectable marker, were selected with WR99210 (de Koning-Ward et al., 2000). Transfections with *pbgst-ko 2A* and *2B* were done in parallel with a previously described plasmid targeting the *pbmrp* as a transfection control (Rijpma et al., 2016).

Integration of knockout plasmids was verified by Southern analysis of chromosomes separated by CHEF or FIGE. Chromosome blocks were prepared as previously described (Serrano et al., 1999). Southern blots were hybridized with specific probes: *pbgst-ko 1* plasmid with the *tgdhfr/ts* probe (921 bp) and *pbgst-ko 2* plasmid with the *hdhfr* probe (774 bp).

### Sequence Alignment and Structural Homology Model of the *P. berghei* GST

GST amino acid sequences from *P. berghei, P. falciparum*, and human were aligned using ClustalW (Thompson et al., 1994; Larkin et al., 2007) with default parameters. Sequence alignment was visualized by GeneDoc (Nicholas et al., 1997); some manual editing was done to produce the final alignment. The PbGST 3D structural homology model was generated using the *P. falciparum* GST (PDB code 1Q4J) (Perbandt et al., 2004) structure as template with the I-TASSER server using default parameters (Roy et al., 2010). The predicted translated sequence of the PbGST protein was identical to the one available at PlasmoDB (PBANKA_102390) and was used to generate the 3D structural homology model. Analysis and visualization of the 3D structural model were performed using PyMOL (DeLano, 2002). The PbGST 3D structural model was superimposed on the PfGST-GSH bound structure (PDB code 3FR9) (Liebau et al., 2009), and G and H binding sites were analyzed. The ConSurf Server^1^ (Ashkenazy et al., 2010, 2016) was used to estimate the conservation profile of G and H binding sites of *P. berghei*, *P. falciparum*, and three human GST orthologs. The structure-based sequence alignment of PbGST, PfGST, and hGST was done with ESPript 3.0 (Robert and Gouet, 2014).

### Structure-Based Screening

**FIGURE 2:**
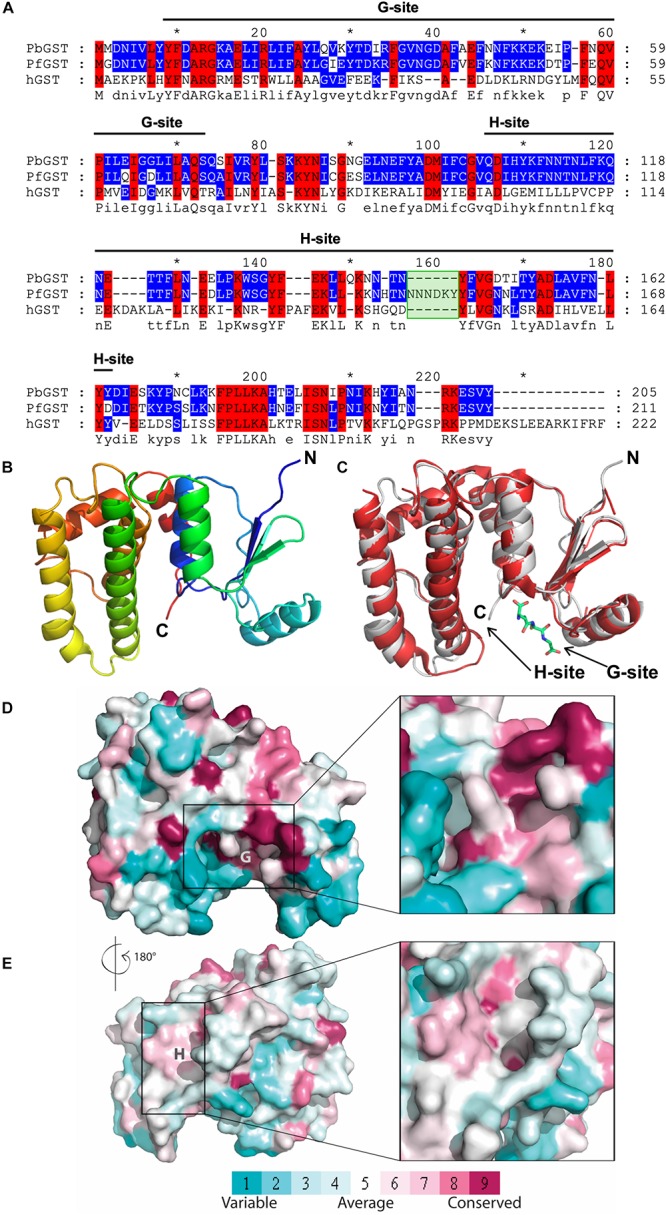
Sequence alignment and homology model of *Plasmodium berghei* GST. **(A)** Sequence alignment of GSTs from *P. berghei*, *P. falciparum*, and human ortholog. GST sequences of *P. falciparum* (PfGST), *P. berghei* (PbGST) and human (hGST) were aligned using ClustalW. Amino acid sequence identity is displayed using discrete colors for user defined range of identities as follows as: primary level of display – 100% conservation (red with black letters); secondary level of display (2 out of three residues conserved, blue with black letters); and a tertiary level of display (no conservation) is displayed as black letters in a white background. Dashes (–) represent gaps inserted between residues to produce an optimal alignment, and the consensus sequence of the alignment is shown at the bottom of the sequence. The six amino acids insertion in the PfGST is indicated as a green box. The black line indicates the corresponding G-site and H-site. **(B)** 3D structural homology model of the PbGST monomer. The PbGST 3D structural model was predicted with the I-TASSER server using the *P. falciparum* GST structure as a template. **(C)** Structural alignment between PbGST model and PfGST. The structural superposition alignment of PbGST model (gray) with the PfGST-GSH bound structure (red) shows a high degree of similarity. The GSH ligand is shown as sticks at the G-site. Arrows indicate the binding sites, G-site and H-site. **(D,E)** Comparison of the G and H binding sites in *P. berghei*, *P. falciparum*, and human GST ortholog and their level of conservation. The GST structure is displayed as a surface conformation, and the G and H binding sites are demarcated with a rectangle. Close-ups of the G and H binding sites are shown on the right panel of the figure. The amino acids are colored by their conservation grades using the color-coding bar in which turquoise through magenta indicates variable through conserved residues. The PDB codes for GSTs used in this figure are 1Q4J for PfGST and three human GST orthologs (1PKZ, 1GTU, and 4EDY). Both the sequence and structural alignments revealed that the G-site is highly conserved, while variability is observed in the H-site of *Plasmodium* spp., and human GSTs.

**FIGURE 3:**
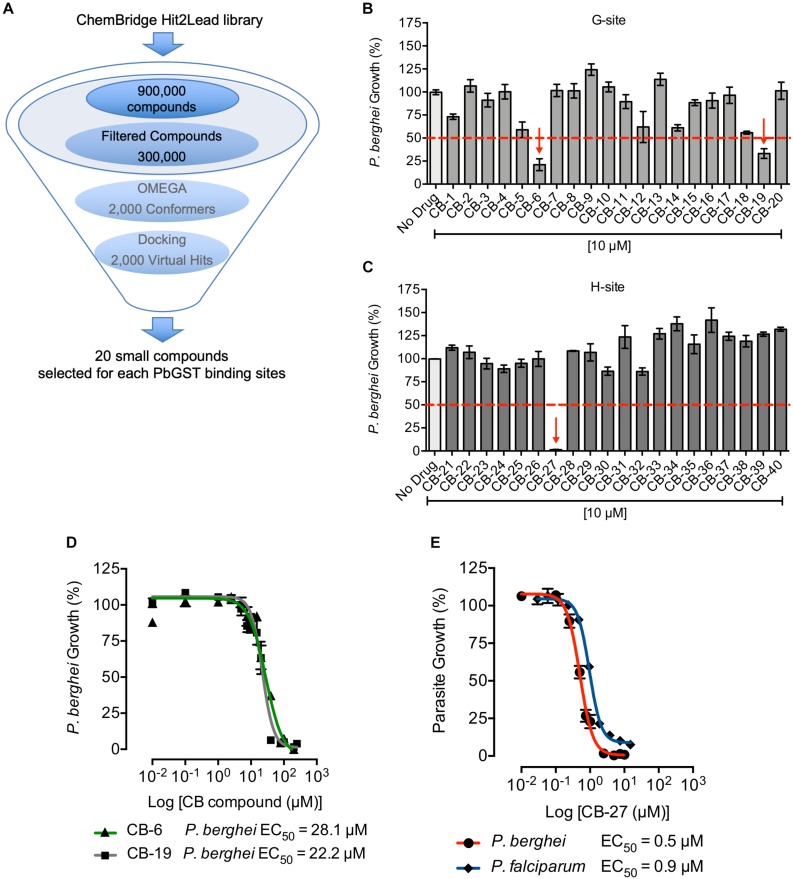
Structure-based screening against PbGST and antiplasmodial activity of CB compounds. **(A)** Structure-based screening pipeline. Steps for the structure-based screening process of the ChemBridge Hit2Lead library to identify potential PbGST inhibitors. **(B,C)** *P. berghei* initial drug screening of selected CB compounds at 10 μM. Red dashed line indicates the 50% inhibition cutoff. A total of three compounds showed > 50% of parasite growth inhibition as indicated by red arrows. Data are means ± SD and represent one biological experiment in triplicate each. **(D)** Dose-response curves of CB-6 and CB-19 in *P. berghei*. CB-6 (green line with triangles) and CB-19 (gray line with squares) showed *P. berghei* growth inhibition at EC_50_ = 28.1 μM (95% CI 20.9–37.8), and EC_50_ = 22.2 μM (95% CI 19.1–25.9), respectively. **(E)** Dose-response curves of CB-27 in *P. berghei* (red line), and multidrug-resistant *P. falciparum* Dd2 clone B2 (blue line). CB-27 showed inhibition of *in vitro* development of *P. berghei* at EC_50_ = 0.505 μM (95% CI 0.463–0.551), and *P. falciparum* Dd2-B2 parasites at EC_50_ = 0.958 μM (95% CI 0.871–1.054). Data are means ± SEM and represents four independent experiments in triplicate each for *P. berghei* and six independent experiments in duplicate each for *P. falciparum* Dd2-B2 parasites.

Structure-based screening of the ChemBridge Hit2Lead library (dataset download on February 21, 2013)^2^ was done using the PbGST 3D structural homology model, taking advantage of the highly conserved binding sites of *P. berghei* and *P. falciparum* (Figures 2D,E). A structure-based method (Figure 3A) was used that applied docking scoring computations and was conducted using a dimer of the PbGST against the two binding sites, G-site and H-site. The OpenEye Scientific software package (Hawkins et al., 2010; Hawkins and Nicholls, 2012; McGann, 2012) was used for docking analysis using standard parameters. Hits were pre-screened for potential liabilities based on known reactive properties of small molecules using the FILTER tool before running the structure-based screening. OMEGA2 was used to generate a maximum of 2,000 conformers for each compound. Docking analyses were done using Fast Rigid Exhaustive Docking (FRED) with standard parameters (McGann, 2012). Docking results were then visualized using VIDA for the following parameters: formation of hydrogen bonds by ligand atoms with residues of the PbGST binding sites, reasonable ligand conformation, and energetically favorable interactions.

### Shape Similarity Screening

Shape similarity screening was done with the Rapid Overlay of Chemical Structures (ROCS) tool (version 3.2.2.2) from OpenEye Scientific software package (Hawkins et al., 2010; Hawkins and Nicholls, 2012; McGann, 2012) and the ChemBridge Hit2Lead library. Multi-conformer files were generated by OMEGA2 and saved in oeb.gz format. Multi-conformational files were used to carry out a ROCS similarity search. ROCS uses a smooth Gaussian function to identify ligands using a shape-based superimposition method to find similar compounds. In addition to the outline of the shape for the query molecule, ROCS can be instructed to follow chemical constrains such as acceptor, donor or hydrophobicity in a certain area of the shape. Known favorable interactions observed in the docking pose of the query molecule can be emphasized in the ROCS search strategy to identify chemically similar molecules derived from a different scaffold. For the analysis, ROCS uses the heavy atoms ignoring the hydrogens. The output files of the shape similarity screening reported rigorous Tanimoto and Tversky measure between shapes and were ranked according to their ROCS combo score based on matching 3D shape and chemistry. The shape similarity screening hops from one chemical scaffold to another to improve potency, selectivity, and physicochemical properties. The hits obtained by shape similarity screening were subjected to molecular docking into the PbGST H-site. Hits were ranked considering the molecular shape, chemistry, and predicted binding interactions into the PbGST H-site.

### Antiplasmodial Activity in *P. berghei* Parasites

The *P. berghei* GFP-Luc_ama__1_ (1037cl1) parasite line was used to assess antiplasmodial activity of ChemBridge Hit2Lead library compounds (CB compounds) and the half-maximal effective concentration (EC_50_) by *in vitro* drug luminescence assay as previously described (Lin et al., 2013). This assay was standardized using chloroquine diphosphate salt (Sigma-Aldrich^®^) as control (100 nM) for complete inhibition of blood stage development. The specific GST inhibitors, S-hexylglutathione and ellagic acid (Sigma-Aldrich^®^) were dissolved in 100% DMSO to prepare the stock solution. CB compounds were purchased in powder form and dissolved in 100% DMSO to obtain a 10 mM stock solution, aliquoted and stored at −20°C. CB dilutions were prepared in RPMI 1640 medium supplemented with 20% heat-inactivated fetal bovine serum (Gibco^®^) and Neomycin stock solution of 10,000 IU/ml (Sigma-Aldrich^®^) within 24 h before initiation of the experiment and stored at 4°C. Initial testing of the compounds was done in triplicate at 10 μM for each. Compounds that inhibited > 50% of parasite growth at 10 μM were chosen for dose-response curve analysis using at least eight compound concentrations. Data analysis was performed as previously described (Lin et al., 2013), and the EC_50_ were calculated using GraphPad Prism 6 software. Dose-response curves for each compound were done in at least four independent experiments each in triplicate.

### Antiplasmodial Activity in *P. falciparum* Multidrug-Resistant Dd2 Clone B2 Parasites

*In vitro* susceptibility to CB-27 was done in the *P. falciparum* multidrug-resistant Dd2 clone B2. Briefly, mostly ring-stage parasites were incubated at 0.2% initial parasitemia and 1% hematocrit with a range of CB-27 concentrations at 37°C for 72 h in 96-well plates as previously described (Ekland et al., 2011). After 72 h, parasite growth was assessed using flow cytometry on an Accuri C6 cytometer with parasites stained with SYBR green I and MitoTracker Deep Red. CB-27 was tested in six independent experiments with technical replicates. The percentage of parasite growth was curve fitted against log-transformed drug concentrations, and the EC_50_ was calculated using GraphPad Prism 6 software.

### Determination of *P. berghei* GST Inhibition

Inhibition of GST was determined in crude *P. berghei* ANKA 507cl1 protein extracts from blood stages. White blood cells were removed using Plasmodipur filters (Euro-Diagnostica) and red blood cells lysed using 0.15% saponin followed by centrifugation. Parasite pellets were resuspended in buffer (3.5 mM MgCl_2_, 110 mM KCl, 40 mM NaCl, 20 mM HEPES, 6 mM EDTA, pH 7.4) with protease inhibitors (0.01 mg of leupeptin A, 0.001 mg of pepstatin A, 0.35 mg of PMSF) lysed by three freeze/thaw cycles (liquid nitrogen and 37°C water bath), and protein content was determined using Bio-Rad *DC* Protein Assay. The GST inhibition assay was standardized using the specific GST inhibitor, S-hexylglutathione, and human placenta GST (Sigma-Aldrich^®^, Cat. No. G8642) as a positive control. Inhibition of PbGST by CB-27 was determined by adding CB-27 (1, 10, and 50 μM) with a 0.65 mg/ml *P. berghei* protein extract in a total volume of 200 μl containing 1 mM of 1-chloro-2,4-dinitrobenzene (Sigma-Aldrich^®^) and 100 mM potassium phosphate buffer (pH 6.5) at 25°C. CB-27 dilutions were prepared in DMSO (0.5% final concentration). The reaction was initiated with GSH (1 mM final concentration), and activity was measured at 340 nm in a 96-well plate (UV flat bottom Microtiter^®^, from Thermo Fisher Scientific) using the SpectraMax M3 Microplate Reader (Molecular Devices). To detect any residual GST activity associated with the parasite extract, blank reactions control (no GSH), experimental control (no extract), and compound control (no extract plus compound at higher concentration) were evaluated. Slope values for activity were obtained directly from the microplate reader control software in the unit of mAU per min and converted to μmol/min by the following formula:

Activity⁢in⁢μ⁢mol/min=[Slope/(ε×b)]×total⁢volume

where ε is the micromolar extinction coefficient for the product S-(2,4-dinitrophenyl)glutathione ε340 nm = 0.0096 μM^–1^cm^–1^, *b* is the path length of 0.89 cm for a total volume of 0.0002 L. The data were analyzed and plotted as GST activity (μmol/min) versus compound concentration.

### Erythrocyte Cell Lysis Assay

The CB compounds identified as lead compounds were analyzed at 10 serial dilutions using fresh mouse erythrocytes at 1% hematocrit in Dulbecco’s PBS (DPBS, Gibco^®^) in V-bottom microplates (Corning^®^ 96 well TC-treated microplate). The plates were incubated for 24 h at 37°C, followed by centrifugation at 2,000 rpm for 5 min, and 50 μl of supernatant was transferred to a fresh flat-bottom microplate (BD Falcon^®^). The amount of hemoglobin released into the supernatant was determined using the QuantiChrom^TM^ Hemoglobin Assay Kit (BioAssay Systems, Cat. No. DIHB-250) according to manufacturer’s instructions. Saponin at 100 μg/ml was used as a positive control for 100% cell lysis, blood (1% hematocrit) with DPBS as a negative control for no cell lysis, and DPBS as a blank. Lead compounds were tested in three independent experiments in triplicate each.

### Pharmacokinetic and Toxicity Parameters of Novel Antiplasmodial Lead Compounds

Pharmacokinetic and toxicity properties including absorption, distribution, metabolism, excretion, and toxicity (ADMET), were predicted using the pkCSM server (Pires et al., 2015)^3^. The pkCSM used the SMILE string to predict ADMET parameters using graph-based signatures to develop predictive regression and classification models. ADMET parameters were verified for compliance with their standard ranges and compared to CQ. Pharmacokinetic and toxicity properties results were analyzed and evaluated, as recommended by Pires et al. (2015).

## Results

### *Plasmodium berghei* Glutathione S-Transferase Gene Is a Target for Antimalarial Drug Discovery

The full-length *pbgst* coding region of *P. berghei* ANKA 507cl1 was amplified by PCR and sequenced. The ANKA 507cl1 *gst* sequence was identical to that previously reported (PBANKA_102390) for ANKA strain, consisting of a genomic and a coding sequence of 808 bp (GenBank accession number: MH794462) and 618 bp (GenBank accession number: MH794463), respectively (Supplementary Figure S1A). Reverse transcription-PCR confirmed the presence of a 109 bp intron and a transcript with two exons of 38 bp and 580 bp (Supplementary Figures S1B,C). The predicted translated DNA sequence revealed a 205 amino acids protein with a predicted molecular weight of 24.04 kDa.

To investigate whether the *P. berghei gst* gene is essential for parasite development in the vertebrate host, we attempted to disrupt the gene in the ANKA 507cl1 reference line (Figure 1). Four independent transfections were carried out using a plasmid designed to disrupt the *pbgst* gene by double crossover recombination (Figures 1A,C). Two out of four transfections selected pyrimethamine-resistant parasites (Figures 1B,D and Supplementary Table S3). Southern blot analysis of DNA from two pyrimethamine-resistant parasites showed no integration into the *pbgst* locus on chromosome 10 (Figures 1B,D). This observation strongly suggests that the *pbgst* gene is essential for parasite development during the intra-erythrocytic stages. To validate this result, an alternative strategy to disrupt the *pbgst* gene was employed using a *pbgst-ko* plasmid with the *hdhfr/yfcu* positive-negative selectable marker (Figure 1E). Two independent transfections were carried out, and resistant parasites were obtained following WR99210 selection (Figures 1F,G and Supplementary Table S3). Southern blot analysis of eight lines from two independent transfections showed no integration of the *pbgst-ko* plasmid into the *pbgst* locus (Figures 1F,G). To confirm transfection efficiency, the unrelated non-essential *P. berghei* multidrug resistance-associated protein (*pbmrp*) gene (Rijpma et al., 2016), was targeted in parallel. Disruption of *pbmrp* was successful, as shown by the integration of the *pbmrp-ko* plasmid into their locus, chromosome 14 (Figures 1F,G).

As a proof of principle, the susceptibility of *P. berghei* blood stages to the specific GST inhibitors S-hexylglutathione and ellagic acid (Supplementary Figures S2A,B) was evaluated. As predicted, the specific GST inhibitors displayed parasite growth inhibition at EC_50_ of 6.9 and 4.4 μM for S-hexylglutathione and ellagic acid, respectively (Supplementary Figure S2C). A chloroquine dose-response curve was used as control (Supplementary Figure S2D). These results show that the specific GST inhibitors block *P. berghei* intra-erythrocytic development and support the essential role of the enzyme as shown by the transfection experiments.

### Identification of CB-27 as a Novel Antiplasmodial Compound Targeting *P. berghei* Glutathione S-Transferase

To identify novel lead compounds that potentially target the malaria parasite GST, we analyzed the sequence and structural properties of *P. berghei* and *P. falciparum* GSTs compared to hGST ortholog. Sequence alignment revealed significant differences in amino acid composition between *Plasmodium’s* enzyme and the hGST (Figure 2A). Alignment of the *Plasmodium* sequences, PbGST and PfGST, shows a high degree of sequence identity (Figure 2A) and a significant difference consisting of a deletion of six amino acids at position 140–145 in the PbGST sequence (green box) (Figure 2A). A PbGST 3D structural homology model was generated by comparative modeling with the PfGST (PDB code 1Q4J) as a template (Figure 2B). As predicted, the structural alignment of PbGST and PfGST showed a high degree of similarity (Figure 2C). Comparison of the G and H binding sites of *P. berghei*, *P. falciparum*, and the three hGST orthologs (PDB codes 1PKZ, 1GTU, and 4EDY) using the ConSurf Server, revealed that the G-site was highly conserved in all GSTs species (Figure 2D, shaded in magenta) while variability in the H-site of *Plasmodium* spp., and hGSTs (Figure 2E) was observed. This variability in the H-site represents a target region for screening of small molecules that potentially exclusively inhibit *Plasmodium* GST empowering the discovery of novel antimalarials.

The PbGST 3D structural homology model was used to perform structure-based screening to identify novel compounds that potentially inhibit the *Plasmodium* GST activity. Results using the ChemBridge Hit2Lead library revealed 2,000 virtual hits that potentially interact with the PbGST G and H binding sites (Figure 3A). Docking of the virtual hits into the PbGST binding sites was evaluated for diverse and low energy conformations. Individual visual inspection of docking results allowed the selection of hits that dock into each binding site, G-site and H-site, and show favorable binding interactions (Supplementary Table S4). Twenty compounds for each binding site displaying the best binding interactions were selected for antiplasmodial activity testing (Supplementary Table S4).

The potential antiplasmodial activity of these 40 compounds identified through structure-based screening was determined using the *P. berghei in vitro* drug luminescence assay (Lin et al., 2013). A cutoff of 10 μM was chosen for the initial screening (Figures 3B,C). Those compounds that produced parasite growth inhibition of > 50% were subsequently analyzed in dose-response curves. Three compounds (CB-6, CB-19, and CB-27, Supplementary Figure S3) were selected for dose-response curves as they inhibited *P. berghei* intra-erythrocytic growth at 10 μM (Figures 3B,C). Results showed that CB-6, CB-19, and CB-27 inhibited *P. berghei* intra-erythrocytic growth with EC_50_ of 28.1, 22.2, and 0.5 μM, respectively (Figures 3D,E). CB-27 had the lowest EC_50_ (0.5 μM) in *P. berghei*, making it an ideal candidate for further studies on *P. falciparum*.

**FIGURE 4:**
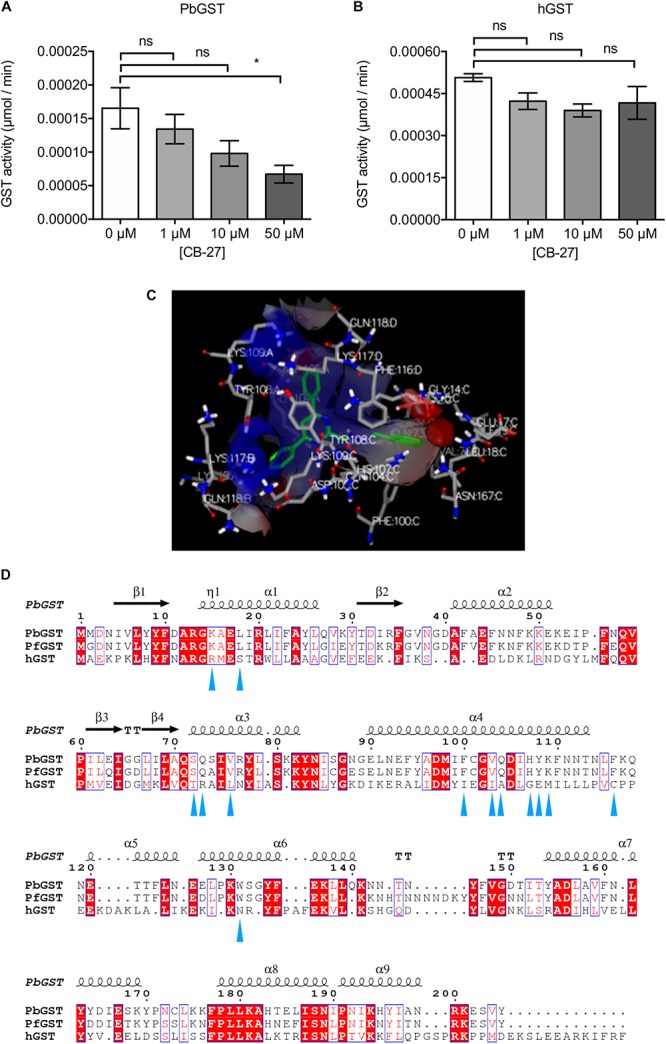
Inhibition of PbGST by CB-27 and proposed binding mode. GST inhibition by CB-27 was determined at three different concentrations (1, 10, and 50 μM) in: **(A)** crude *P. berghei* protein extracts from blood stages, and **(B)** human placenta GST. Data are means ± SEM and represents four independent experiments. PbGST activity was significantly reduced at 50 μM (^∗^*p* < 0.0418), analyzed by One-Way ANOVA with Bonferroni’s multiple comparisons test. **(C)** Predicted binding mode and interactions of CB-27 in the PbGST H-site. The structure of the PbGST is shown as a silhouette with the binding site displayed as a surface representation. CB-27 is represented as sticks with carbon (green), and nitrogen (blue). The amino acids of the protein are represented as sticks with carbon (gray), hydrogen (white), nitrogen (blue), and oxygen (red). Amino acid residues interacting in the proximity of the compounds are displayed with three-letter code, and the number represents the position in the polypeptide. **(D)** Structure-based sequence alignment of PbGST, PfGST, and hGST. Key residues interacting with CB-27 are highlighted with blue triangles below the sequence.

A dose-response curve of CB-27 carried out in synchronized multidrug-resistant *P. falciparum* Dd2 clone B2 parasites, revealed an EC_50_ of 0.9 μM (Figure 3E). Furthermore, *P. berghei* and *P. falciparum* showed similar response to CB-27 on blood stage parasites. The potential inhibition of GST enzyme activity by CB-27 was analyzed using GST inhibitor S-hexylglutathione as a positive control for PbGST and hGST assays (Supplementary Figures S4A,B). A dose-dependent inhibition of the PbGST was observed with increasing concentrations of CB-27, suggesting that the compound blocks GST enzyme activity (Figure 4A). CB-27 did not inhibit hGST in the same assay (Figure 4B). These results support the hypothesis that CB-27 targets *Plasmodium* GST activity specifically.

Docking simulation of CB-27 at the PbGST H-site predicted a strong interaction (Figure 4C). The predicted key residues interacting with CB-27 in *P. falciparum*, *P. berghei*, and human GSTs are shown in Figure 4D. In *Plasmodium* GSTs, the key residues are located at the alpha helix 4 which consists of two hydrophobic (phenylalanine, F; valine, V), and four polar amino acids (glutamine, Q; histidine, H; tyrosine, Y; lysine, K). The polar amino acids are predicted to mediate CB-27 binding into PbGST H-site. The specific inhibitory activity observed against PbGST can be the result of the different key residues between *Plasmodium* and human GSTs.

### Identification of Six Novel Antiplasmodial Lead Compounds by CB-27 Shape Similarity Screening

**FIGURE 5:**
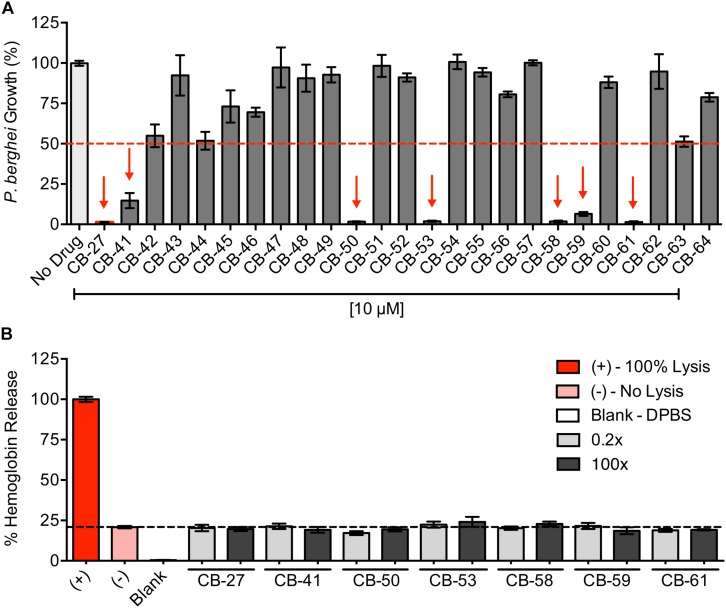
Antiplasmodial activity of CB compounds identified by CB-27 shape similarity screening and potential hemolytic activity of the novel lead compounds. **(A)** *P. berghei* initial drug screening of 24 compounds and CB-27 at 10 μM. The 50% inhibition cutoff is indicated by the red dashed line. Red arrows highlight six compounds that showed > 50% of parasite growth inhibition. Data are means ± SD and represent one biological experiment in triplicate each. **(B)** Analysis of potential hemolytic activity of the novel antiplasmodial lead compounds. Experimental controls include the following: positive control – saponin at 100 μg/ml for 100% cell lysis; negative control – blood with DPBS (1% hematocrit) for no cell lysis; blank – DPBS. All lead compounds were tested at ten serial dilutions from 0.2X to 100X fold of their EC_50_ value from antiplasmodial dose-response curves, but only 0.2X and 100X are shown in the graph. Data are means ± SEM and represents three independent experiments in triplicate each. Novel antiplasmodial lead compounds show no lytic activity against erythrocytes.

CB-27 is a novel chemical scaffold with antiplasmodial activity making it an ideal candidate to be used in a shape similarity screening to identify analogs. Identified hits from the shape similarity screening were ranked according to their ROCS combo score (Supplementary Table S5). The 24 top-ranked hits with ROCS combo scores from 1.93 to 1.29 were selected considering their molecular shape, chemistry, electrostatic parameters, and the predicted binding interactions into the more diverse PbGST H-site (Supplementary Figure S5). Analysis for antiplasmodial activity revealed six novel chemical scaffolds, CB-41, CB-50, CB-53, CB-58, CB-59, and CB-61, which inhibited *P. berghei* intra-erythrocytic growth at 10 μM (Figure 5A). Dose-response curves of these compounds showed inhibition of *P. berghei in vitro* development ranging from 0.6 to 4.9 μM (Table 1 and Supplementary Figure S6).

**TABLE 1:** Calculated EC_50_ values and 95% confidence interval of the novel lead compounds from the antiplasmodial dose-response curves.

**Compound**	**EC_50_**	**95% CI**
CB-27	0.5	0.46–0.5
CB-41	4.9	4.4–5.3
CB-50	1.3	1.2–1.5
CB-53	0.8	0.7–0.8
CB-58	1.1	1.0–1.2
CB-59	1.1	0.9–1.4
CB-61	0.6	0.5–0.6

### Pharmacokinetic and Toxicity Parameters of Novel Antiplasmodial Lead Compounds

Early evaluation of drug-likeness helps select the best leads during drug discovery and development. To investigate the drug-likeness features of the seven lead compounds identified herein, predicted ADMET parameters were determined using pkCSM (Pires et al., 2015). The pharmacokinetic and toxicity properties of the lead compounds (CB-27, CB-41, CB-50, CB-53, CB-58, CB-59, and CB-61) were compared to CQ (Supplementary Table S6).

We calculated parameters associated with absorption such as water solubility, membrane permeability in colon cancer cell line (Caco2), intestinal absorption, skin permeability levels, and P-glycoprotein substrate or inhibitor (Pgp subs, Pgp I/II inh). The seven lead compounds were predicted to be water-soluble with values within -3.554 to -4.913 log mol/L, similar to the predicted value of CQ (-4.249 log mol/L). Caco2 permeability is considered high when Papp coefficient is > 8 × 10^–6^, and the predicted value is > 0.90. Therefore, CB-27 and CB-59 were predicted to have high Caco2 permeability. Compounds displaying absorbance of less than 30% are considered to have reduced intestinal absorption. The lead compounds were predicted to have high absorption with estimated values that ranged from 88.9 to 100%, similar to CQ (89.95%). Skin permeability is vital for transdermal drug delivery, and a log Kp > -2.5 is considered to have relatively low skin permeability. Like CQ, all lead compounds were predicted to be skin permeable. The lead compounds and CQ were predicted to be both P-glycoprotein (PgP) substrates and PgP I/II inhibitors.

Four predictors influence drug distribution, including volume of distribution (VDss), fraction unbound, blood-brain barrier (BBB) permeability, and Central Nervous System (CNS) permeability. The VDss is the theoretical volume that a drug needs to be uniformly distributed to produce the same plasma concentration. The VDss is estimated low when log VDss < -0.15 and high when log VDss > 0.45. Except for CB-50 and CB-59, the other leads had an estimated low VDss (Supplementary Table S6). CB-27 and CB-59 had predicted values for unbound fraction of 0.185 and 0.237, respectively; which are similar to CQ (0.191). BBB permeability, and CNS permeability can estimate drug distribution into the brain. Compounds with log BB > 0.3 are suggested to cross readily the BBB while compounds with log BB < -1 cross poorly. Like CQ, all lead compounds were predicted to cross the BBB with values that ranged from -0.702 to 0.143 log BB. The blood-brain permeability-surface area product (log PS) measures CNS permeability in which compounds with a log PS > -2 are suggested to penetrate the CNS while those with log PS < -3 are unable to penetrate the CNS. All lead compounds were predicted to penetrate the CNS.

Drug metabolism was predicted based on the CYP models for substrate or inhibition. Results show that the lead compounds are predicted substrates for CYP3A4 and were predicted to inhibit the isoenzymes CYP2C19, and CYP3A4 (Supplementary Table S6). Drug excretion was measured using two predictors, the renal OCT2 substrate predictor that describes the potential of a drug to be secreted by the kidney, and total clearance that combines hepatic clearance and renal clearance. The predicted renal OCT2 data (Supplementary Table S6) suggest that the lead compounds are non-substrates of the OCT2 pathway. Differences in predicted total clearance can be observed between lead compounds with estimated values that range from -0.257 to 0.84 log ml/min/kg. Total clearance predictions show all lead compounds with a lower total clearance than CQ and, that of the lead compounds, CB-27 has the predicted highest total clearance followed by CB-41, CB-53, and CB-58.

The AMES test (carcinogenicity), hERG inhibition (cardiotoxicity), hepatotoxicity, and skin sensitization were used to predict the toxicity of the lead compounds. According to AMES toxicity prediction, CB-50, CB-53, and CB-59 are not mutagenic. However, CQ (Chatterjee et al., 1998) and CB-27, CB-41, CB-58, and CB-61 have a predicted positive AMES test (Supplementary Table S6). The predictions suggest that all lead compounds and CQ are hERG II inhibitors (Supplementary Table S6). The predictions suggest that all lead compounds and CQ may have hepatotoxic potential but do not cause skin sensitization.

The lead compounds were shown to inhibit *P. berghei* intra-erythrocytic growth. We then investigated their potential effects on the erythrocytes by measuring the hemoglobin release. Erythrocyte lysis potential was evaluated at ten serial dilutions starting at 0.2-100X fold above their EC_50_ against parasites. None of the lead compounds hemolyzed erythrocytes even at the highest (100X) concentrations (Figure 5B), showing that the antiplasmodial effects of the lead compounds are not due to toxicity against erythrocytes.

Overall, the lead compounds displayed predicted ADMET properties similar to CQ, suggesting favorable disposition of the compounds in the organism. Based on pharmacokinetic and toxicity predictions, the lead compounds should be further developed. Both analyses, the predicted ADMET and the erythrocyte lysis assay, provide excellent insights for further studies in their development as antimalarials.

## Discussion

Malaria is one of the most critical public health challenges worldwide due to the emergence of multidrug resistance. The discovery of novel effective antimalarials is crucial for treatment and disease control with eradication as the goal. Herein, we demonstrate that the *pbgst* gene is refractory to disruption, therefore considered a validated drug target. Structure-based screening followed by biological and biochemical testing identified CB-27 as a novel antiplasmodial lead compound targeting PbGST. Through structure-based screening against the PbGST model, CB-27 shape similarity screening and biological testing, six additional lead compounds were discovered that inhibit *P. berghei* intra-erythrocytic growth with EC_50_ of 0.6–4.9 μM. These compounds represent novel leads for antimalarial drug discovery and development.

Genes encoding enzymes in the *Plasmodium* glutathione biosynthetic pathway are critical for parasite development and represent potential antimalarial targets for novel drugs (Vega-Rodríguez et al., 2009; Buchholz et al., 2010; Pastrana-Mena et al., 2010; Patzewitz et al., 2012). We previously demonstrated that *P. berghei* gamma-glutamylcysteine synthetase and glutathione reductase genes are dispensable for intra-erythrocytic growth but crucial for parasite development in the mosquito, arresting development at the oocyst stage (Vega-Rodríguez et al., 2009; Pastrana-Mena et al., 2010). In contrast, *P. falciparum* gamma-glutamylcysteine synthetase and glutathione reductase genes are known to be essential for the intra-erythrocytic stages (Patzewitz et al., 2012; Zhang et al., 2018). Likewise, *Plasmodium* GST, a multifunctional cytosolic enzyme that mediates cellular detoxification (Harwaldt et al., 2002; Liebau, 2002; Deponte and Becker, 2005; Liebau et al., 2005) has been proposed as a drug target (Harwaldt et al., 2002; Fritz-Wolf et al., 2003). Five attempts to disrupt the gene using two knockout plasmids with different selectable markers were made (Figure 1) to determine whether the *pbgst* gene is essential. Our results (Figure 1) show that the cytosolic GST of *P. berghei* is refractory to genetic disruption and essential for parasite intra-erythrocytic development; thus, an attractive target for drug development. These results are in agreement with recent reports showing that *P. falciparum gst* is an essential non-mutable gene (Zhang et al., 2018), confirming its validity as a drug target and a new avenue in searching for novel antimalarials. However, a recent report using large-scale gene knockout study suggested that the *pbgst* gene (PBANKA_1023900) is dispensable (Bushell et al., 2017). These divergent data might be the result of *P. berghei* mutants that were generated by a large-scale genetic screen, and the genotype of this mutant was not confirmed (RMgmDB database: RMgm-2830).

**FIGURE 6:**
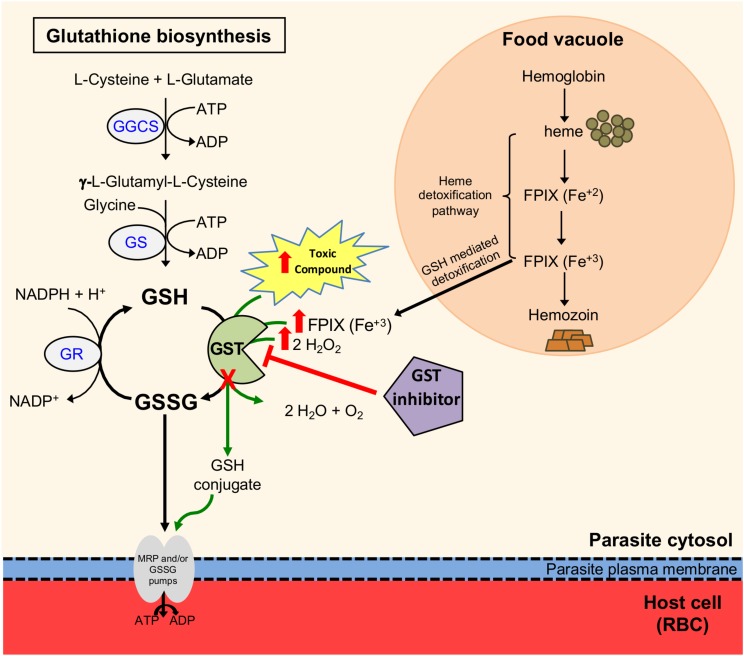
Predicted model of disruption or inhibition of the glutathione S-transferase in *P. berghei* intra-erythrocytic stages. The diagram shows the glutathione (GSH) redox system in *P. berghei* intra-erythrocytic stages. GSH is recycled by glutathione reductase (GR) and glutathione S-transferase (GST), which maintain the balance between GSH and oxidized glutathione (GSSG). GST functions include: GSH conjugation to toxic compounds, GSH mediated detoxification of ferriprotoporphyrin IX (FPIX) where GST serves as a ligandin and detoxification of hydrogen peroxide (depicted as green arrows). GST conjugates GSH to a variety of compounds containing electrophilic centers forming GSH conjugates that are recognized and exported by multidrug resistance-associated protein (MRP) and/or GSSG pumps. Hemoglobin digestion in the food vacuole forms heme, which is converted into hemozoin crystals through the heme detoxification pathway. A portion of heme is transported to the parasite’s cytosol where it is conjugated to GSH by GST for its detoxification. Genetic or chemical disruption of PbGST interferes with their detoxification, resulting in accumulation of toxic compounds, including H_2_O_2_ and FPIX (represented by red arrows), leading to parasite death.

Additional reports based on biological studies found that GST inhibitors, such as ellagic acid, S-hexylglutathione, among others, are active against *P. falciparum* and *P. vinckei petteri* pointing out their antimalarial action (Srivastava et al., 1999; Harwaldt et al., 2002; Soh et al., 2009; Sturm et al., 2009). Similar to previous results, our data show that S-hexylglutathione and ellagic acid, previously reported as GST inhibitors, hinder *P. berghei* intra-erythrocytic growth. Moreover, *Plasmodium* GST inhibitors have been reported to synergize with CQ, presumably by blocking FPIX detoxification (Srivastava et al., 1999; Harwaldt et al., 2002; Soh et al., 2009). Collectively, these results support the predicted model that disruption or inhibition of *Plasmodium* GST will stop parasite development potentially via interference with detoxification, resulting in the accumulation of toxic compounds like FPIX and increasing oxidative stress leading to parasite death (Figure 6).

This study used a comprehensive structure-based computational approach to discover potential PbGST inhibitors in the ChemBridge Hit2Lead library (Muegge, 2003; Ursu et al., 2011; Kar and Roy, 2013; Yusof and Segall, 2013; Lionta et al., 2014; Ferreira et al., 2015; Kontoyianni, 2017). Based on structure-based sequence alignment, the amino acid composition of the G and H sites, and molecular docking simulations it was predicted that hits binding to the PbGST binding sites would also bind to the PfGST binding sites. From the experimental evaluation of the 40 prospective hits emerged one promising compound, CB-27, that inhibited intra-erythrocytic growth of *P. berghei* at EC_50_ of 0.5 μM. Similarly, CB-27 also inhibited the growth of the multidrug-resistant *P. falciparum* Dd2 clone B2 at EC_50_ of 0.9 μM. Molecular docking simulations predict that CB-27 bind to the H-site of PbGST, potentially inhibiting the enzyme. It is of interest to note that *Plasmodium* GST H-site differs significantly from the hGST ortholog (Figure 4D). To experimentally assess whether CB-27 is a GST inhibitor; an enzymatic inhibition assay was conducted using *P. berghei* crude protein extracts showing dose-dependent inhibition of GST activity. The hGST ortholog was not inhibited, positioning CB-27 as a promising compound that binds and inhibits the *Plasmodium* GST. These results support the notion that CB-27 is acting via inhibition of the GST detoxification system. It is of interest to note that the ability of CB-27 to inhibit parasite growth in culture is more potent than its ability to inhibit the GST enzyme from crude protein extract (EC_50_ = 0.5 μM and IC_50_ > 10 μM, respectively). This apparent discrepancy is not uncommon for this type of compound as similar results have been previously reported for ellagic acid, which displays 100 fold higher potency against parasites than against the purified GST enzyme, with a Ki of 74 μM in an enzyme assay but an EC_50_ of 0.8 μM in antiparasitic assays (Sturm et al., 2009). Potential explanations for these discrepancies are off-target effects of the compounds as well as the use physiological GSH concentrations (1 mM) but non-physiological CDNB concentrations (1 mM) in the corresponding enzyme assays. The values obtained under such high “drug” concentrations are likely to be distorted.

Shape similarity screening is widely used in early drug development to discover novel chemical scaffolds and to optimize the potency and pharmacokinetic properties of the lead compounds (Kumar and Zhang, 2015). CB-27 was used as query in a shape similarity screening to identify other chemical scaffolds. The rationale is that two compounds with similar shape and chemistry are likely to fit into the same binding site and will display similar biological activity. The CB-27 shape similarity screening resulted in 24 hits that were tested in an *in vitro* drug susceptibility assay. Compounds CB-41, CB-50, CB-53, CB-58, CB-59, and CB-61 exhibited antiplasmodial activity against *P. berghei* at low micromolar concentrations (EC_50_ of 0.6–4.9 μM), demonstrating the advantage of using this approach to discover new chemical scaffolds. These results showed that CB-27 shape similarity screening allowed for the identification of a group of novel antiplasmodial lead compounds.

All lead compounds inhibit *P. berghei* intra-erythrocytic growth, and we showed that none induce hemolysis (Figure 5B). The hemolytic activity analysis revealed that these lead compounds are not toxic to erythrocytes. Poor pharmacokinetic properties are a leading cause of failure in the drug development process (Page et al., 2016). In the early stages of drug discovery and development, the prediction of pharmacokinetic and toxicity properties has become a practical approach to facilitate prioritization of potential hits, and lead optimization. All lead compounds are predicted to fulfill the absorption requirements. The predicted differences between the lead compounds and CQ in P-glycoprotein modulation, and CYP2D6 and CYP3A metabolism suggests that the lead compounds present favorable and less metabolism-based drug interactions. Drug distribution predictors, BBB permeability and CNS permeability, suggest that the lead compounds can cross the BBB comparable to CQ (Adelusi and Salako, 1982) and supports its potential use to treat cerebral malaria. According to drug excretion predictions, CB-27 has the highest total clearance, and all lead compounds are not renal OCT2 substrates. The successful use of lead compounds depends on their toxicity, and predictions suggest no differences between CQ and the lead compounds in terms of inhibition of hERG I/II, and hepatotoxicity. These lead compounds have drug-like properties with acceptable pharmacokinetic profiles for oral route due to their predictions of high intestinal absorption, metabolism in the liver, drug distribution into the brain, and low excretion. Results from pharmacokinetic and toxicity predictions suggest that ADMET profiles are similar to CQ. The pharmacokinetic and toxicity prediction, in conjunction with the erythrocyte lysis assay, support further studies on the development of these lead compounds as antimalarials.

In summary, we are reporting novel lead compounds with antiplasmodial activity which offer potential scaffolds for the development of antimalarial agents. These compounds derive from a structure-based screening of 900,000 compounds from ChemBridge Hit2Lead library, followed by CB-27 shape similarity screening. The identified lead compounds represent novel structures with antiplasmodial activity not previously reported. Further medicinal chemistry optimization to improve structure activity relationship and potency as well as safety and toxicity, pharmacokinetic (bioavailability, half-life and biodistribution) and rodent *in vivo* studies will follow to determine the compounds potential to become suitable candidates for further development. Future studies will also be required to assess the effect that GST amino acid variations between species (i.e., human and *Plasmodium*) could have on the compound efficacy against this target.

## Data Availability Statement

The datasets generated for this study can be found in the GenBank accession numbers: MH794462 and MH794463.

## Ethics Statement

The animal study was reviewed and approved by the Institutional Animal Care and Use Committee (Protocol number 2480108) at the AAALAC accredited Animal Resources Center of the University of Puerto Rico-Medical Sciences Campus.

## Author Contributions

EC-L, JV-R, JB, and AS conceived and designed the experiments. EC-L, DC-L, JV-R, AD, and JB conducted the experiments. EC-L, DC-L, JV-R, AD, DF, AB-O, JO, JB, and AS analyzed the data. DF, JB, and AS contributed the reagents, materials, and analysis tools. EC-L and AS wrote the manuscript. JV-R, DF, AB-O, and JB revised the manuscript critically. All authors approved the final manuscript.

## Conflict of Interest

JB is the co-founder of InterRayBio, LLC. The remaining authors declare that the research was conducted in the absence of any commercial or financial relationships that could be construed as a potential conflict of interest.

## Footnotes

1 http://consurf.tau.ac.il

2 http://www.hit2lead.com

3 http://biosig.unimelb.edu.au/pkcsm/prediction
