# Supplementary material for: Structure-Based Screening of Plasmodium berghei Glutathione S-Transferase Identifies CB-27 as a Novel Antiplasmodial Compound
Source: Front Pharmacol. 2020 Mar 17;11:246. doi: 10.3389/fphar.2020.00246 (PMC7090221; doi:10.3389/fphar.2020.00246)
Supplement: Supplementary file 1 [file Data_Sheet_1.docx]

Supplementary Material

**SUPPLEMENTARY TABLE S1 |** Primers used for *P. berghei gst* sequencing.

| **Primer #** | **Nucleotide Sequence (5’ 🡪 3’)** | **Base Pairs** | **Sense (S) / Antisense (AS)** |
| --- | --- | --- | --- |
| 211 | GGGATGATGGACAACATAGTGCTG | 24 | S |
| 212 | CCCGAATATCTTGTACACCAC | 21 | AS |
| 213 | CCCGGTGATGCATTTGCAGAATTTAAC | 27 | S |
| 214 | CCCTTAATAGACGCTTTCTTTTCTATTAGC | 30 | AS |
| 215 | CCCCTTAATAGACGCTTTCTTTTC | 24 | AS |

**SUPPLEMENTARY TABLE S2 |** Primers used for *P. berghei gst* knockout plasmids.

| ***pbgst-ko* Plasmid**  (backbone plasmid) | **Primer #** | **Nucleotide Sequence (5’ 🡪 3’)**  (Restriction sites underlined) | **Size** | **Sense (S) / Antisense (AS)** | **Restriction Site** | **Region** | **Fragment Size** |
| --- | --- | --- | --- | --- | --- | --- | --- |
| ***pbgst-ko 1A***  (pL0001) | 64 | GGGGGTACCAGATCTGCTATACTTAAAATGATGGACAACATAGTGC | 46 bp | S | KpnI | 5’ UTR *pbgst* | 638 bp |
|  | 65 | GGGAGGCCTAAGCTTCAAAATAACCTGACCATTTAGGTAATTCTTC | 46 bp | AS | HindIII |  |  |
|  | 68 | GGGGGTACCGGATCCGGGGATACTATAACATATGCAGATTTAGCAG | 46 bp | S | BamHI | 3’ UTR *pbgst* | 553 bp |
|  | 69 | GGGGATATCTCTAGAGCACATATTATATATGTATGTATATACAATGCTC | 49 bp | AS | XbaI |  |  |
| ***pbgst-ko 1B***  (pL0001) | 193 | GGGGGTACCCTTAGTTAATCTGAAAGTATATGTTAATAAC | 40 bp | S | KpnI | 5’ UTR *pbgst* | 586 bp |
|  | 194 | GGGAAGCTTGCGGAAATAAAATTACATAACACACAATG | 38 bp | AS | HindIII |  |  |
|  | 195 | GGGGGATCCTAGCAAAATAGTATAGTATTATTCTGTTTG | 39 bp | S | BamHI | 3’ UTR *pbgst* | 700 bp |
|  | 196 | GGGTCTAGATCATAATGACACACATTCAAAAATAAGGC | 38 bp | AS | XbaI |  |  |
| ***pbgst-ko 2A***  (pL0034) | 270 | CCGCGGGTTACCATTACCCAGAGTTCAC | 28 bp | S | SacII | 5’ UTR *pbgst* | 556 bp |
|  | 271 | CTGCAGCAGCACTATGTTGTCCATC | 25 bp | AS | PstI |  |  |
|  | 272 | GATATCCCATTATTAAAAGCCCATACTG | 28 bp | S | EcoRV | 3’ UTR *pbgst* | 654 bp |
|  | 273 | GAATTCGTGTGCGCAGATATGTATAAGC | 28 bp | AS | EcoRI |  |  |
| ***pbgst-ko 2B***  (pL0034) | 274 | CCGCGGCCCACGTTATTTAATAGTTTTAGTTACC | 34 bp | S | SacII | 5’ UTR *pbgst* | 576 bp |
|  | 275 | CTGCAGGCACTATGTTGTCCATCATTTTAAG | 31 bp | AS | PstI |  |  |
|  | 276 | GATATCCATTATATTGCTAATAGAAAAGAAAGCGTC | 36 bp | S | EcoRV | 3’ UTR *pbgst* | 486 bp |
|  | 277 | GAATTCGTTTACTATAAATCACTTATTTTCTG | 32 bp | AS | EcoRI |  |  |

**SUPPLEMENTARY TABLE S3 |** *P. berghei gst* gene disruption experiments.

| **Knockout Plasmid** | **Transfection #** | **Samples #** | **Outcome** | **Attempts** |
| --- | --- | --- | --- | --- |
| *pbgst-ko 1A* | 1 | 2 | No integration | 1 |
| *pbgst-ko 1B* | 2 | 2 | No parasite | 3 |
|  | 3 | 2 | No parasite |  |
|  | 4 | 1 | No integration |  |
| *pbgst-ko 2A* | 5 | 2 | No integration | 2 |
|  | 6 | 2 | No integration |  |
| *pbgst-ko 2B* | 5 | 2 | No integration | 2 |
|  | 6 | 2 | No integration |  |

**SUPPLEMENTARY TABLE S4 |** Compounds identified through structure-based screening of the ChemBridge Hit2Lead library against PbGST binding sites. Compounds are listed with laboratory ID (Lab ID), Hit2Lead library Identification (Hit2Lead ID), molecular weight (g/mol), predicted binding site and docking score. Docking analyses were scored using the Chemgauss 4 scoring functions.

| Lab ID | Hit2Lead ID | g/mol | Binding Site | Docking score |
| --- | --- | --- | --- | --- |
| CB-1 | 47731832 | 397.5 | G-site | -9.6979 |
| CB-2 | 75621646 | 438.6 | G-site | -9.1567 |
| CB-3 | 37012934 | 357.5 | G-site | -9.0084 |
| CB-4 | 6373314 | 412.5 | G-site | -9.7125 |
| CB-5 | 6073008 | 361.4 | G-site | -8.6675 |
| CB-6 | 6147495 | 499.6 | G-site | -8.5721 |
| CB-7 | 6372703 | 398.5 | G-site | -8.3073 |
| CB-8 | 6378442 | 398.5 | G-site | -8.1264 |
| CB-9 | 6627346 | 408.5 | G-site | -7.8427 |
| CB-10 | 6692265 | 355.4 | G-site | -8.3947 |
| CB-11 | 5216131 | 543.5 | G-site | -8.0503 |
| CB-12 | 5233322 | 452.5 | G-site | -7.1563 |
| CB-13 | 7886932 | 373.8 | G-site | -7.7669 |
| CB-14 | 7917962 | 418.9 | G-site | -7.3435 |
| CB-15 | 7859744 | 464.6 | G-site | -7.2967 |
| CB-16 | 7888930 | 492.6 | G-site | -6.9912 |
| CB-17 | 7853725 | 371.3 | G-site | -6.4278 |
| CB-18 | 7949994 | 416.5 | G-site | -6.0457 |
| CB-19 | 9131201 | 429.9 | G-site | -6.7879 |
| CB-20 | 9298783 | 378.4 | G-site | -7.4946 |
| CB-21 | 7914213 | 473.5 | H-site | -15.9 |
| CB-22 | 7660053 | 461.5 | H-site | -16.57 |
| CB-23 | 7914204 | 433.4 | H-site | -16.68 |
| CB-24 | 7978575 | 474.5 | H-site | -18.55 |
| CB-25 | 7972691 | 462.5 | H-site | -16.74 |
| CB-26 | 6361981 | 403.5 | H-site | -15.94 |
| CB-27 | 5363105 | 444.5 | H-site | -16.13 |
| CB-28 | 6320180 | 442.5 | H-site | -16.15 |
| CB-29 | 7673107 | 417.6 | H-site | -16.57 |
| CB-30 | 5762254 | 393.4 | H-site | -16.28 |
| CB-31 | 6869343 | 447.6 | H-site | -17.38 |
| CB-32 | 6926060 | 439.5 | H-site | -16.25 |
| CB-33 | 7658494 | 506.6 | H-site | -13.65 |
| CB-34 | 7664050 | 419.4 | H-site | -15.84 |
| CB-35 | 7644755 | 472.5 | H-site | -16.41 |
| CB-36 | 5118299 | 382.5 | H-site | -13.45 |
| CB-37 | 5156412 | 340.5 | H-site | -14.61 |
| CB-38 | 5220996 | 428.4 | H-site | -16.53 |
| CB-39 | 5228957 | 314.4 | H-site | -12.18 |
| CB-40 | 5228961 | 320.4 | H-site | -12.94 |

**SUPPLEMENTARY TABLE S5 |** ChemBridge Hit2Lead library compounds identified through shape similarity using CB-27 as shape query. Compounds are listed with laboratory ID (Lab ID), Hit2Lead library Identification (Hit2Lead ID), molecular weight (g/mol) and ROCS combo score.

| Lab ID | Hit2Lead ID | g/mol | ROCS Combo Score |
| --- | --- | --- | --- |
| CB-41 | 5341759 | 424.5 | 1.9300 |
| CB-42 | 5341757 | 408.5 | 1.6750 |
| CB-43 | 5764326 | 396.4 | 1.6110 |
| CB-44 | 5760384 | 408.5 | 1.5370 |
| CB-45 | 5215629 | 379.4 | 1.5640 |
| CB-46 | 5470348 | 413.4 | 1.4820 |
| CB-47 | 6228703 | 432.9 | 1.5250 |
| CB-48 | 6177958 | 402.5 | 1.3480 |
| CB-49 | 7674023 | 411.5 | 1.3340 |
| CB-50 | 5667711 | 490.3 | 1.3250 |
| CB-51 | 7673107 | 417.6 | 1.3700 |
| CB-52 | 7963629 | 434.5 | 1.3170 |
| CB-53 | 5784720 | 367.4 | 1.3370 |
| CB-54 | 7963283 | 453.9 | 1.3200 |
| CB-55 | 6146749 | 423.5 | 1.2690 |
| CB-56 | 5555166 | 381.4 | 1.2990 |
| CB-57 | 6869343 | 447.6 | 1.3180 |
| CB-58 | 5786029 | 397.4 | 1.2920 |
| CB-59 | 5674317 | 440.9 | 1.3360 |
| CB-60 | 5353390 | 396.5 | 1.3360 |
| CB-61 | 5113018 | 380.3 | 1.3060 |
| CB-62 | 5212213 | 504.6 | 1.3480 |
| CB-63 | 5248700 | 353.4 | 1.2990 |
| CB-64 | 5215628 | 412.9 | 1.2900 |

**SUPPLEMENTARY TABLE S6 |** Predicted pharmacokinetic and toxicity properties of the novel antimalarial lead compounds. The lead compounds from ChemBridge Hit2Lead library are listed with the laboratory ID. Chloroquine used as control is listed as CQ. The pkCSM model generated 7 predictors for absorption, 4 predictors for distribution, 7 predictors for metabolism, 2 predictors for excretion, and 10 predictors for toxicity.

| **Parameters** | **Predictors** | **CQ** | **CB-27** | **CB-41** | **CB-50** | **CB-53** | **CB-58** | **CB-59** | **CB-61** | **Unit** |
| --- | --- | --- | --- | --- | --- | --- | --- | --- | --- | --- |
| **Absorption** | Water solubility | -4.249 | -4.627 | -4.913 | -4.229 | -4.891 | -4.729 | -3.554 | -4.675 | log mol/L |
|  | Caco2 | 1.624 | 0.875 | 0.533 | 0.605 | 0.538 | 0.546 | 0.985 | -0.22 | log Papp |
|  | Intestinal abs | 89.95 | 94.826 | 100 | 88.904 | 93.649 | 94.756 | 94.135 | 90.3 | % Absorbed |
|  | Skin perm | -2.679 | -2.735 | -2.734 | -2.737 | -2.737 | -2.739 | -2.735 | -2.739 | log Kp |
|  | Pgp subs | Yes | Yes | Yes | Yes | Yes | Yes | Yes | Yes | Yes/No |
|  | Pgp I inh | No | Yes | Yes | Yes | Yes | Yes | Yes | Yes | Yes/No |
|  | Pgp II inh | No | Yes | Yes | Yes | Yes | Yes | Yes | Yes | Yes/No |
| **Distribution** | VDss | 1.332 | -0.279 | -0.428 | 0.127 | -0.427 | -0.417 | -0.065 | -0.48 | log L/kg |
|  | Fraction unbound | 0.191 | 0.185 | 0.02 | 0 | 0 | 0 | 0.237 | 0 | Fu |
|  | BBB perm | 0.349 | 0.143 | -0.17 | -0.702 | -0.138 | -0.35 | -0.333 | -0.659 | log BB |
|  | CNS perm | -2.191 | -1.719 | -2.153 | -2.08 | -1.775 | -1.946 | -1.569 | -2.381 | log PS |
| **Metabolism** | CYP2D6 subs | Yes | No | No | No | No | No | No | No | Yes/No |
|  | CYP3A4 subs | Yes | Yes | Yes | Yes | Yes | Yes | Yes | Yes | Yes/No |
|  | CYP1A2 inh | No | Yes | No | No | Yes | Yes | Yes | Yes | Yes/No |
|  | CYP2C19 inh | No | Yes | Yes | Yes | Yes | Yes | Yes | Yes | Yes/No |
|  | CYP2C9 inh | No | Yes | Yes | Yes | Yes | Yes | Yes | No | Yes/No |
|  | CYP2D6 inh | Yes | No | No | No | No | No | No | No | Yes/No |
|  | CYP3A4 inh | No | Yes | Yes | Yes | Yes | Yes | Yes | Yes | Yes/No |
| **Excretion** | Total clearance | 1.092 | 0.84 | 0.777 | -0.257 | 0.646 | 0.59 | 0.319 | 0.297 | log ml/min/kg |
|  | Renal OCT2 subs | Yes | No | No | No | No | No | No | No | Yes/No |
| **Toxicity** | AMES | Yes | Yes | Yes | No | No | Yes | No | Yes | Yes/No |
|  | Max tol dose | -0.167 | 0.664 | 0.334 | -0.105 | 0.147 | 0.205 | 0.747 | -0.122 | log mg/kg/day |
|  | hERG I inh | No | No | No | No | No | No | No | No | Yes/No |
|  | hERG II inh | Yes | Yes | Yes | Yes | Yes | Yes | Yes | Yes | Yes/No |
|  | Oral rat LD50 | 2.85 | 3.781 | 2.909 | 2.239 | 2.474 | 2.456 | 3.186 | 2.6 | mol/kg |
|  | Oral rat LOAEL | 1.026 | 0.424 | 1.884 | 2.84 | 2.363 | 2.482 | 1.559 | 2.998 | log mg/kg_bw/day |
|  | Hepatotoxicity | Yes | Yes | Yes | Yes | Yes | Yes | Yes | Yes | Yes/No |
|  | Skin sens | No | No | No | No | No | No | No | No | Yes/No |
|  | *T. pyriformis* | 1.558 | 0.285 | 0.291 | 0.388 | 0.326 | 0.31 | 0.285 | 0.326 | Numeric (log ug/L) |
|  | Minnow | 0.747 | -1.52 | -1.59 | 0.34 | -0.944 | -1.032 | -1.435 | 0.304 | Numeric (log mM) |


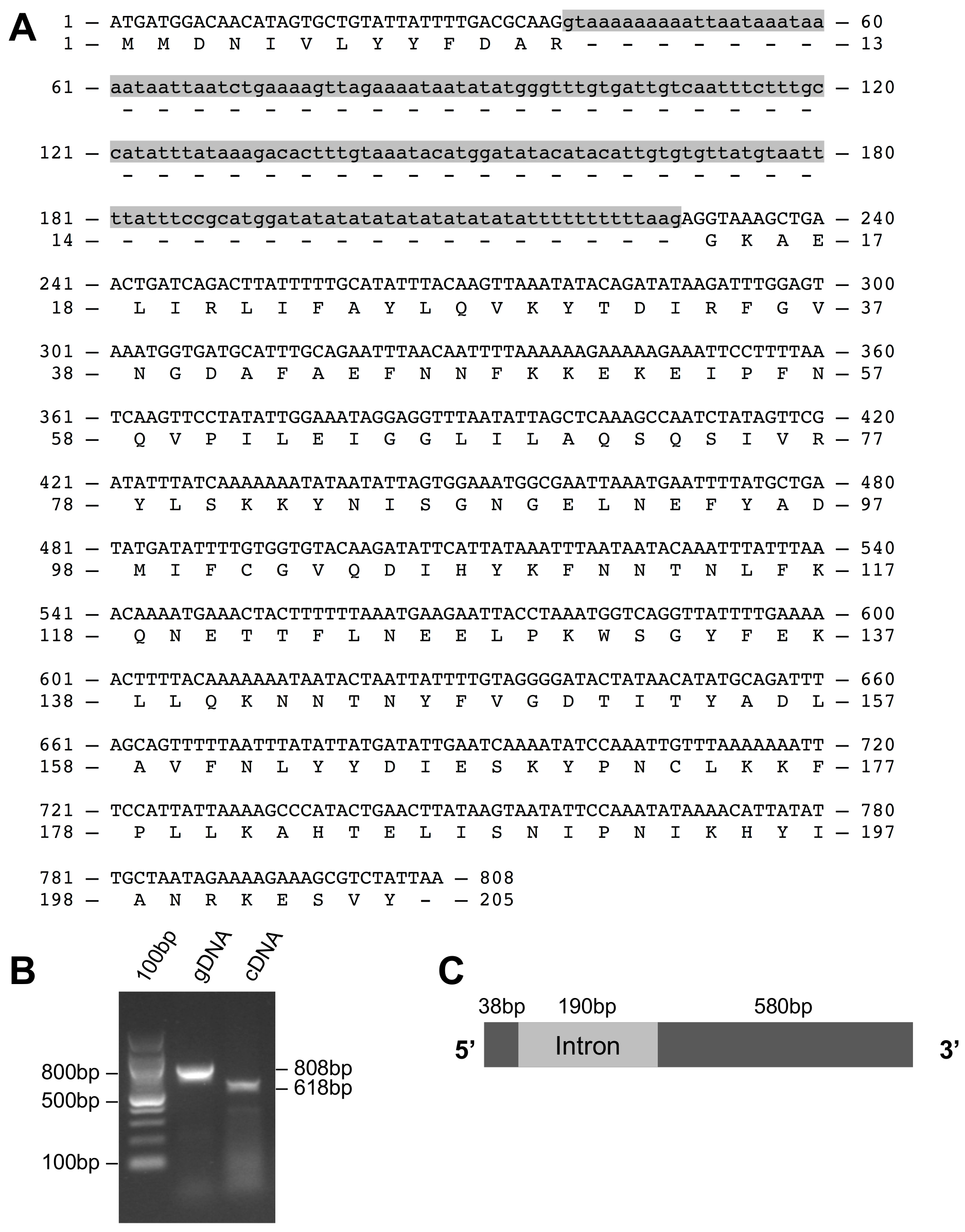


**SUPPLEMENTARY FIGURE S1 |** *Plasmodium* *berghei* glutathione S-transferase gene and protein expression. **(A)** *P.* *berghei* *gst* nucleotide and predicted amino acid sequences. The *pbgst* gene was sequenced using a PCR strategy and consists of 808 nucleotides (GenBank accession number: MH794462). Non-coding regions (intron) are shown by lowercase letters shaded in gray and the coding regions in uppercase letters. The *pbgst* coding sequence consists of 618 nucleotides (GenBank accession number: MH794463). The predicted amino acid sequence is represented as one letter code in uppercase letters and comprises 205 amino acids. **(B)** *P. berghei* gDNA and cDNA were amplified by conventional PCR and Reverse Transcription PCR (RT-PCR) respectively, using primers flanking the coding region of the *pbgst* gene. The size of the PCR products from gDNA (808 bp) and cDNA (618bp) is shown on the right side of the gel. **(C)** Diagram of the *pbgst* gene structure. Exons are shown in gray and the intron in light gray. The size of each region is indicated in base pair (bp) above the corresponding region.

**
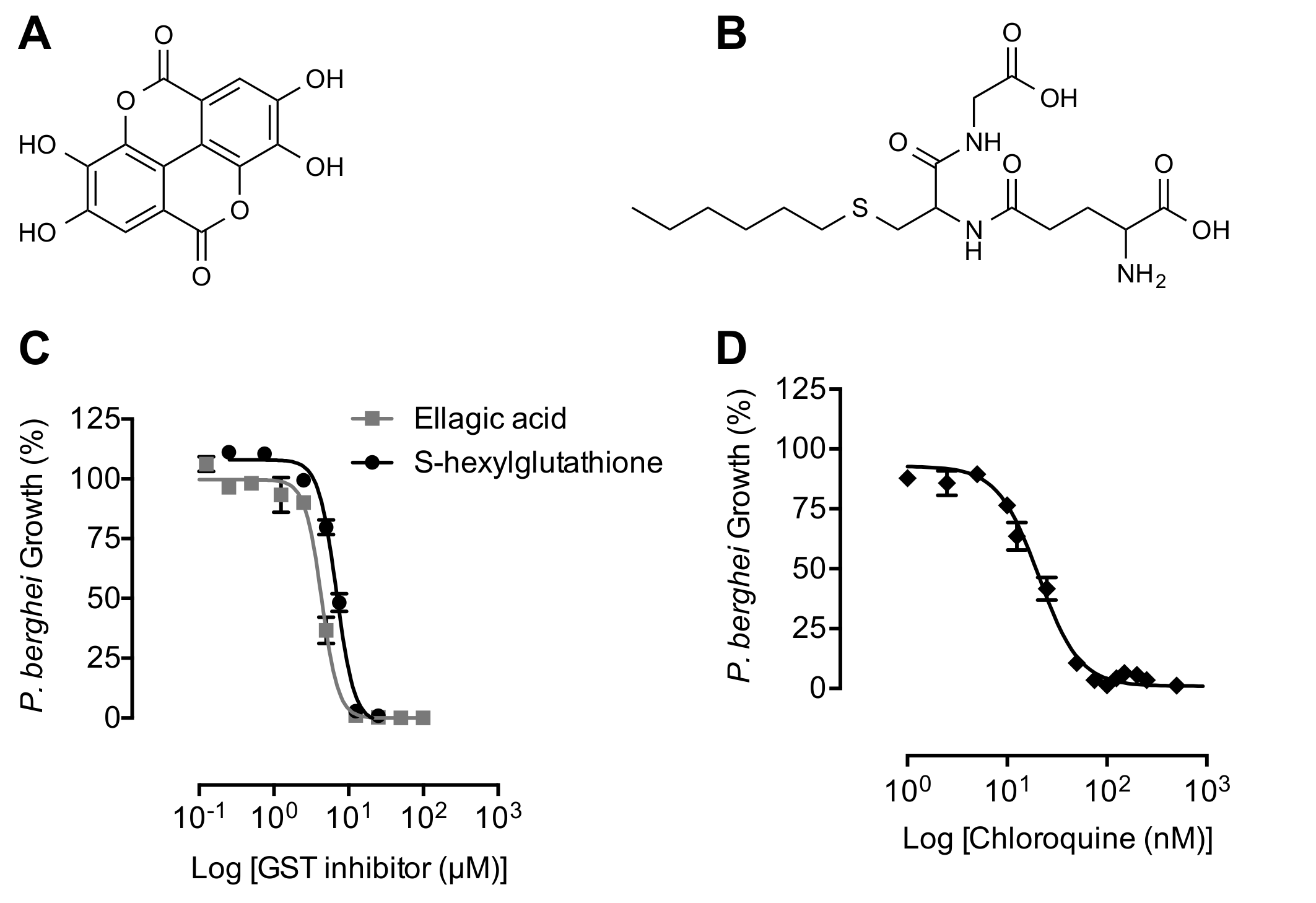
**

**SUPPLEMENTARY FIGURE S2 |** Susceptibility of *P. berghei* to specific GST inhibitors and chloroquine. Chemical structures of the specific GST inhibitors: **(A)** ellagic acid, and **(B)** S-hexylglutathione. **(C)** Inhibition of the *in vitro* development of *P. berghei* blood stages by specific GST inhibitors, ellagic acid (gray squares) and S-hexylglutathione (black circles). Data are means ± SEM and represents at least three independent experiments in triplicate each. Ellagic acid EC_50_ = 4.36 µM (95% CI 4.04 to 4.70). S-hexylglutathione EC_50_ = 6.86 µM (95% CI 6.57 to 7.17). The chemical structures of the compounds are shown. **(D)** Dose response curve of chloroquine. Data are means ± SEM and represents four independent experiments in triplicate each. Chloroquine EC_50_ = 20.70 nM (95% CI 18.74 to 22.86), consistent with published results (EC_50_ = 20 nM) (Lin et al., 2013).


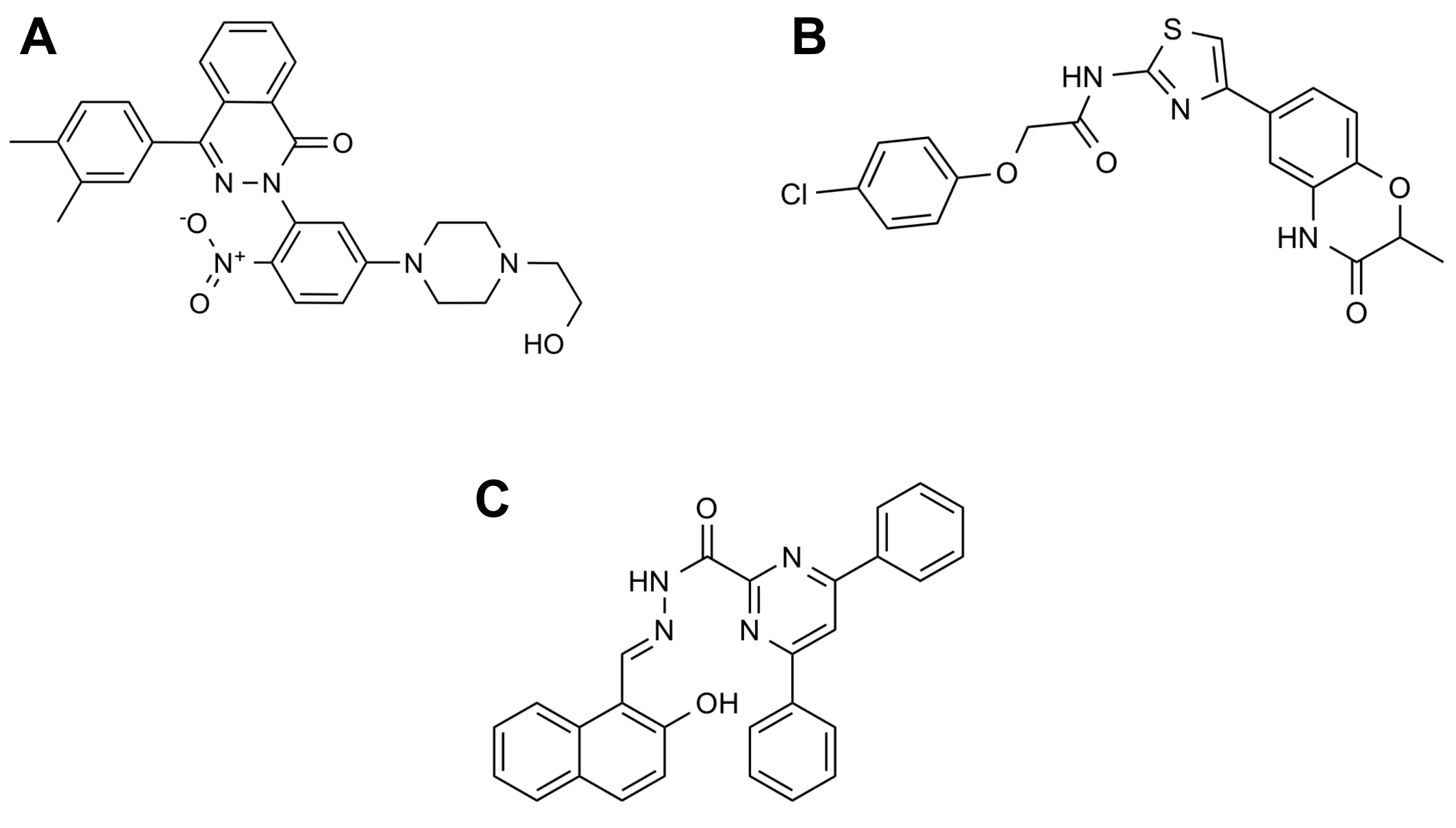


**SUPPLEMENTARY FIGURE S3 |** Chemical structures of CB-6 **(A)**, CB-19 **(B)**, and CB-27 **(C)** compounds.

**
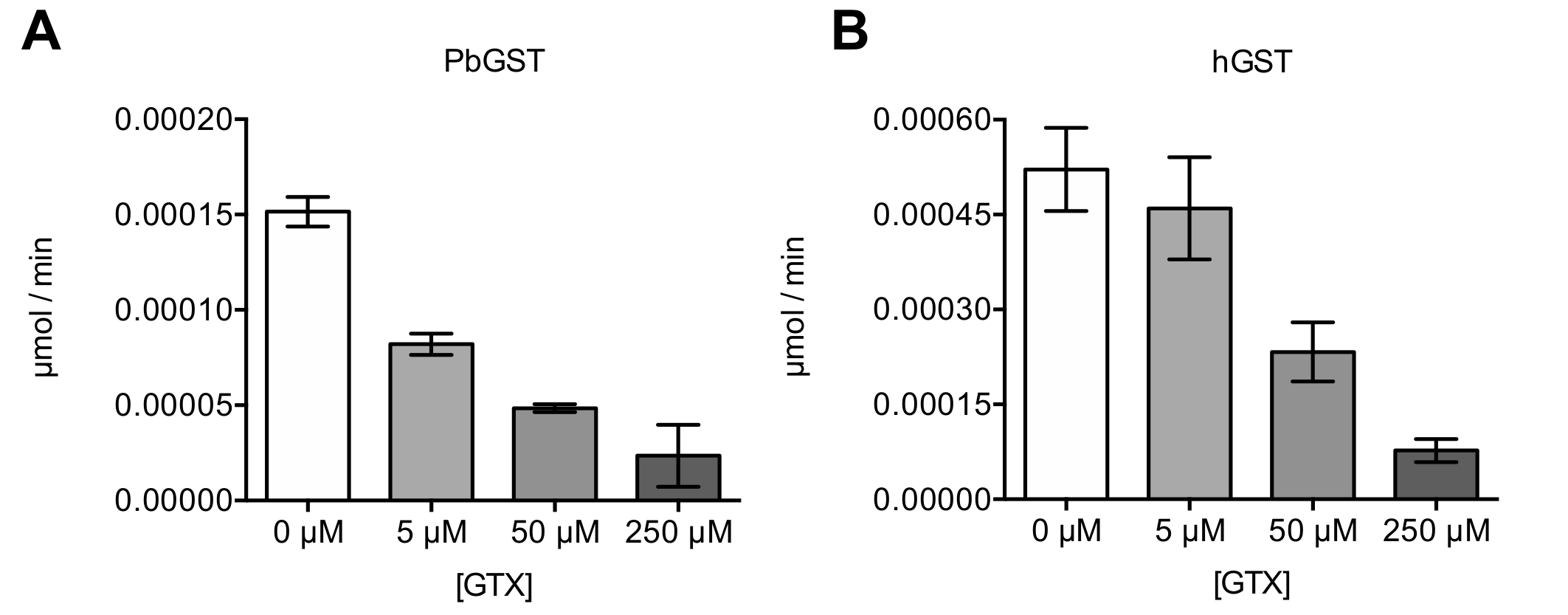
**

**SUPPLEMENTARY FIGURE S4 |** Inhibition of PbGST and hGST activity by the specific GST inhibitor, S-hexylglutathione. As a positive control, the GST inhibition was determined in the presence of a specific GST inhibitor, S-hexylglutathione (GTX), at three different concentrations (5, 50, and 250 µM) in: **(A)** a crude *P. berghei* protein extracts from blood stages, and **(B)** human placenta GST. Data are means ± SD and represents two independent experiments. PbGST and hGST activities were reduced by the inhibitor S-hexylglutathione.


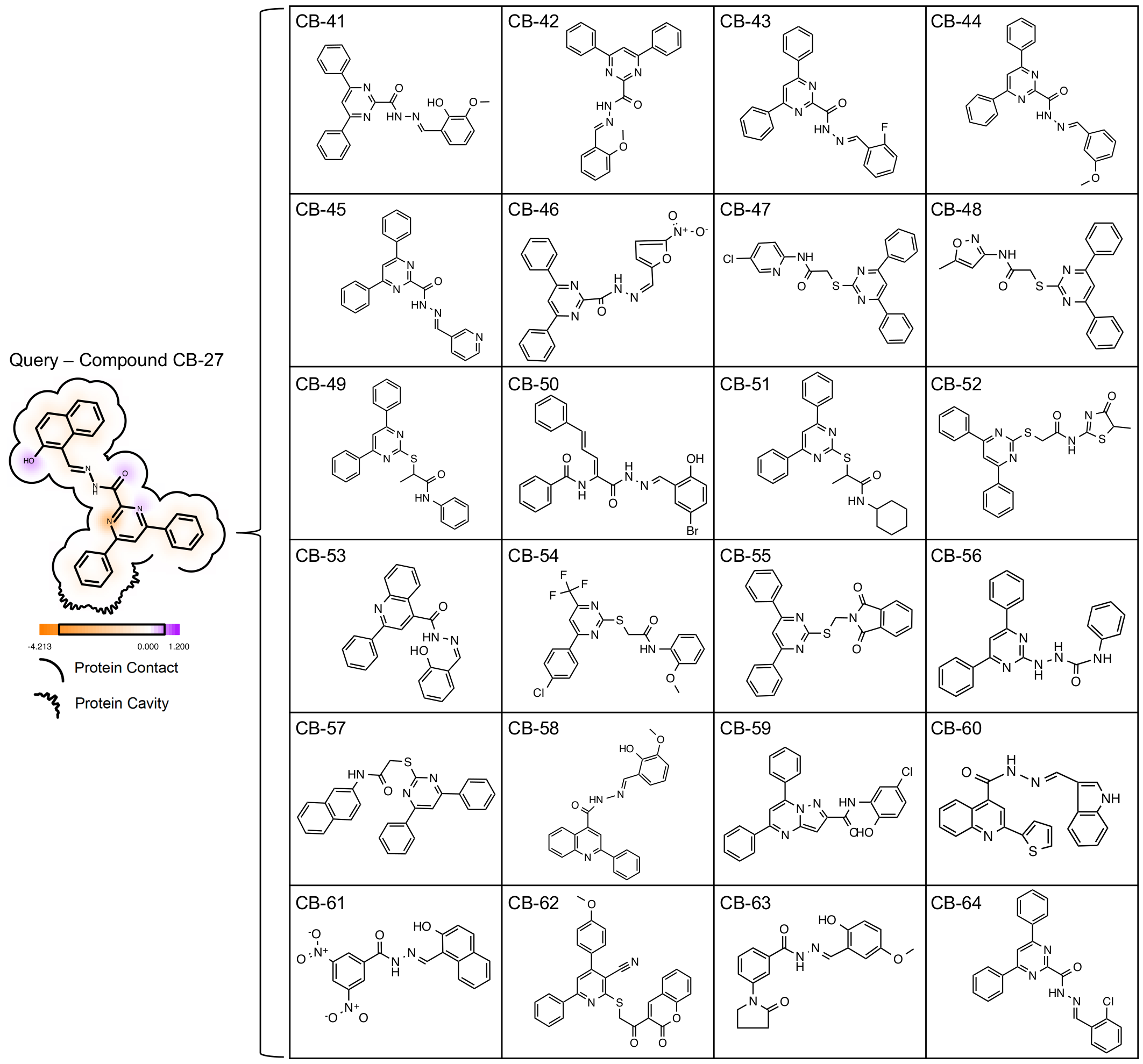


**SUPPLEMENTARY FIGURE S5** | Hits identified by CB-27 shape similarity screening. CB-27, is represented as a 2D ligand-protein complex surface on PbGST. The external arc segments represent the atom accessible to protein contact, and the external eyelash arc segments correspond to the atom accessible to the protein cavity. The colors in the CB-27 structure represent a breakdown of the score by atom. The binding poses of CB-27 into the PbGST H-site were used as templates for the shape and chemical complementarity search in order to identify other chemical scaffolds capable of fulfilling the shape and chemistry. A total of 24 compounds were identified with similar shape and electrostatic properties to CB-27.


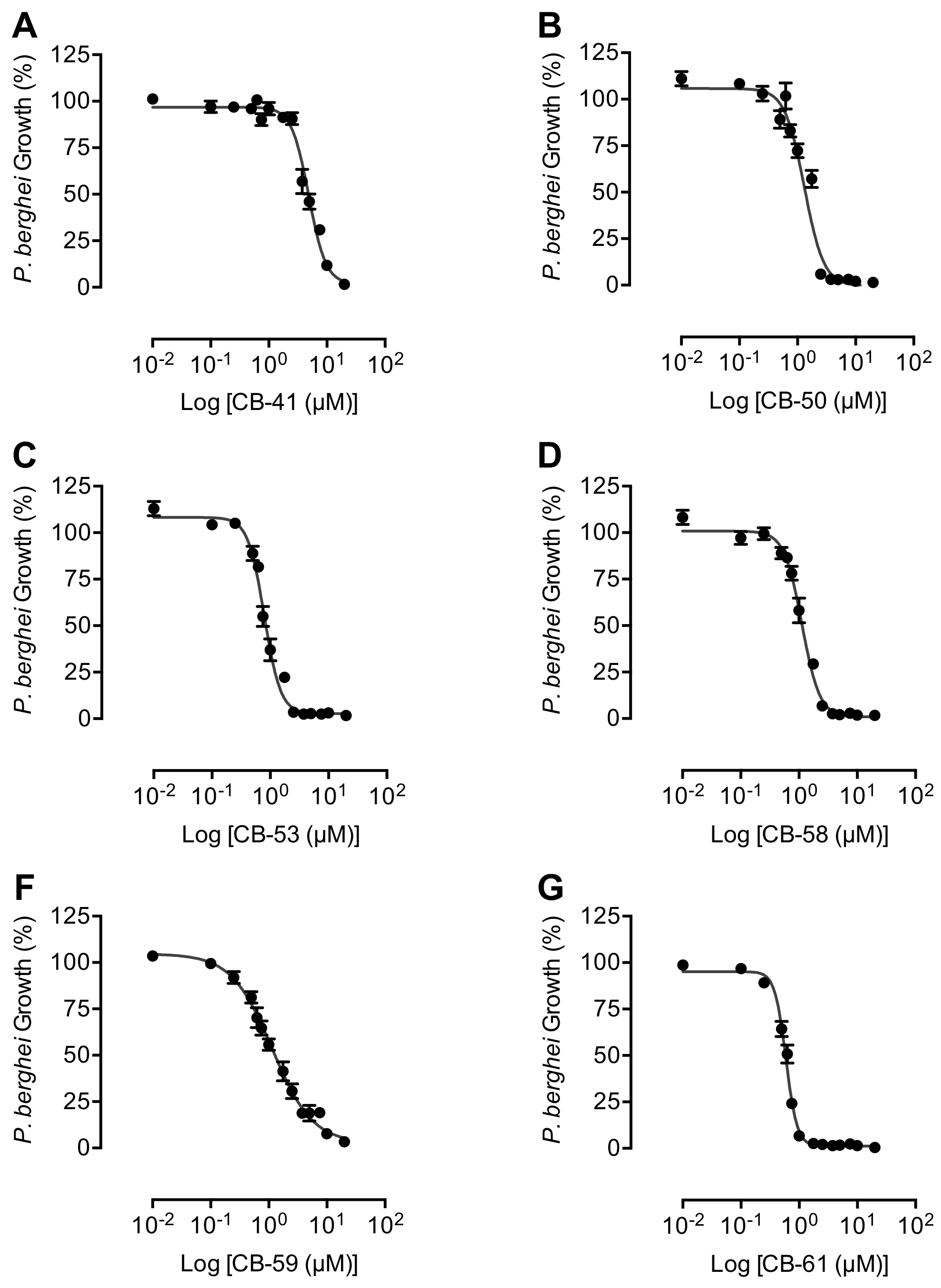


**SUPPLEMENTARY FIGURE S6** | Dose-response curves of the lead compounds identified by CB-27 shape similarity screening. Six novel compounds inhibited parasite growth that ranged from 0.6 µM to 4.9 µM. Data are means ± SEM and represents four independent experiments in triplicate each.

**References**

Lin, J., Sajid, M., Ramesar, J., Khan, S. M., Janse, C. J., and Franke-Fayard, B. (2013). Screening inhibitors of P. berghei blood stages using bioluminescent reporter parasites. *Methods Mol Biol* 923, 507–22. doi:10.1007/978-1-62703-026-7_35.
